# Perilipin 2 downregulation in **β** cells impairs insulin secretion under nutritional stress and damages mitochondria

**DOI:** 10.1172/jci.insight.144341

**Published:** 2021-05-10

**Authors:** Akansha Mishra, Siming Liu, Joseph Promes, Mikako Harata, William Sivitz, Brian Fink, Gourav Bhardwaj, Brian T. O’Neill, Chen Kang, Rajan Sah, Stefan Strack, Samuel Stephens, Timothy King, Laura Jackson, Andrew S. Greenberg, Frederick Anokye-Danso, Rexford S. Ahima, James Ankrum, Yumi Imai

**Affiliations:** 1 Department of Internal Medicine, Carver College of Medicine, University of Iowa, Iowa City, Iowa, USA.; 2 Fraternal Order of Eagles Diabetes Research Center, University of Iowa, Iowa City, Iowa, USA.; 3 Iowa City Veterans Affairs Medical Center, Iowa City, Iowa, USA.; 4 Department of Internal Medicine, Washington University School of Medicine, St. Louis, Missouri, USA.; 5 Department of Neuroscience and Pharmacology, Iowa Neuroscience Institute, University of Iowa, Iowa City, Iowa, USA.; 6 Department of Internal Medicine, Eastern Virginia Medical School, Norfolk, Virginia, USA.; 7 Obesity and Metabolism Laboratory, Jean Mayer United States Department of Agriculture Human Nutrition Research Center on Aging at Tufts University, Boston, Massachusetts, USA.; 8 Department of Medicine, Johns Hopkins School of Medicine, Baltimore, Maryland, USA.; 9 Roy J. Carver Department of Biomedical Engineering, University of Iowa, Iowa City, Iowa, USA.

**Keywords:** Endocrinology, Metabolism, Diabetes, Islet cells

## Abstract

Perilipin 2 (PLIN2) is a lipid droplet (LD) protein in β cells that increases under nutritional stress. Downregulation of PLIN2 is often sufficient to reduce LD accumulation. To determine whether PLIN2 positively or negatively affects β cell function under nutritional stress, PLIN2 was downregulated in mouse β cells, INS1 cells, and human islet cells. β Cell–specific deletion of PLIN2 in mice on a high-fat diet reduced glucose-stimulated insulin secretion (GSIS) in vivo and in vitro. Downregulation of PLIN2 in INS1 cells blunted GSIS after 24-hour incubation with 0.2 mM palmitic acid. Downregulation of PLIN2 in human pseudoislets cultured at 5.6 mM glucose impaired both phases of GSIS, indicating that PLIN2 is critical for GSIS. Downregulation of PLIN2 decreased specific OXPHOS proteins in all 3 models and reduced oxygen consumption rates in INS1 cells and mouse islets. Moreover, we found that PLIN2-deficient INS1 cells increased the distribution of a fluorescent oleic acid analog to mitochondria and showed signs of mitochondrial stress, as indicated by susceptibility to fragmentation and alterations of acyl-carnitines and glucose metabolites. Collectively, PLIN2 in β cells has an important role in preserving insulin secretion, β cell metabolism, and mitochondrial function under nutritional stress.

## Graphical abstract

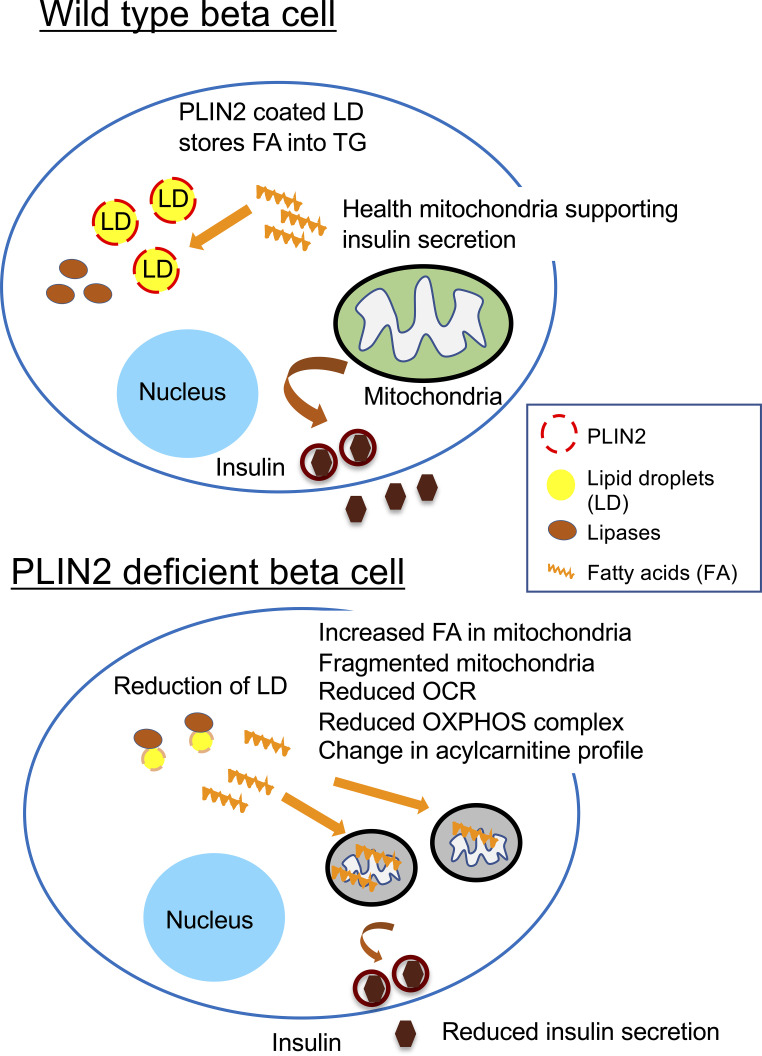

## Introduction

It is widely accepted that the progressive loss of functional β cell mass is a key pathology of type 2 diabetes (T2D) (1, 2). Nutritional stress due to increased influx of glucose and lipids is considered to trigger and accelerate β cell dysfunction in T2D by activating stress pathways, including endoplasmic reticulum (ER) stress, oxidative stress, mitochondrial dysfunction, and inflammation (3, 4). Thus, it is important to understand how β cells respond to an increase in nutritional influx. One consequence of nutritional overload is the increased production of lipids including triglycerides (TG) that are stored in lipid droplets (LDs) (5). While the accumulation of LDs is closely tied to tissue dysfunction in nonalcoholic fatty liver disease, insulin-resistant skeletal muscle, atherosclerosis, and diabetic cardiomyopathy, a role of LD under nutritional stress in β cell dysfunction has been underappreciated due to difficulty demonstrating LDs in mouse β cells (4, 6, 7). Importantly, LDs are easily identifiable in adult human β cells (6, 8, 9). Active formation of LDs in human β cells was shown not only in vitro (9), but also in vivo utilizing human islet transplantation in immunodeficient mice on high-fat diets (HFD) (10). However, it is currently unknown whether LDs in β cells are merely a marker of nutritional overload or whether they play a role in β cell function under physiology and nutritional stress.

The perilipin (PLIN) family of proteins reside on the surface of LDs and regulate intracellular lipid metabolism by controlling the LD formation and mobilization, as well as the interaction with other organelles (11). Among PLINs, PLIN2 is the most abundant PLIN in many nonadipocytes, including β cells (4). We previously demonstrated that PLIN2 is increased in parallel to TG accumulation in islets of mice fed HFD, in *ob/ob* mice, and in human islets after fatty acid (FA) loading (12); these are findings also reported by others (8, 13). Recently, an increase in immunostaining of PLIN2 was reported in human T2D islets, implicating PLIN2 as a possible key PLIN in human T2D (14). PLIN2’s role is not limited to passively affecting lipid accumulation. Rather, PLIN2 actively regulates LD accumulation in a β cell model; the downregulation of PLIN2 by antisense oligonucleotides (ASO) decreased while overexpression of PLIN2 increased FA incorporation into TG in MIN6 cells (9, 12). Thus, the downregulation of PLIN2 should allow us to address whether LDs play a positive or negative role during the development of β cell failure under nutritional stress. Previously, whole body PLIN2–KO mice (exon 2–3 deletion, Δ2–3) carrying Akita insulin mutation demonstrated better preservation of β cell mass and reduced ER stress, suggesting that PLIN2 may protect β cells under extreme ER stress (13). Although ER stress likely has an important role in β cell demise in T2D (15), it remains unknown whether PLIN2 in β cells accelerates or prevents β cell dysfunction under nutritional stress. Considering that downregulation of PLIN2 in MIN6 cells under regular culture conditions impaired acute augmentation of insulin secretion by palmitic acids (PA) (12), it is important to address whether the reduction of PLIN2 improves β cell function under nutritional stress.

Here, we demonstrate that β cell–specific deletion of PLIN2 (exon 5 deletion, Δ5) caused the impairment of insulin secretion both in vivo and in vitro in mice on HFDs. Impairment in insulin secretion was also seen in INS1 cells and human pseudoislets after the downregulation of PLIN2, indicating that PLIN2 supports insulin secretion, especially under nutritional overload. Increased targeting of FA to mitochondria with associated mitochondrial dysfunction was implicated as the basis for β cell dysfunction after PLIN2 downregulation.

## Results

### Insulin secretion is impaired in β cell–specific PLIN2-KO mice on HFD.

**Figure 1:**
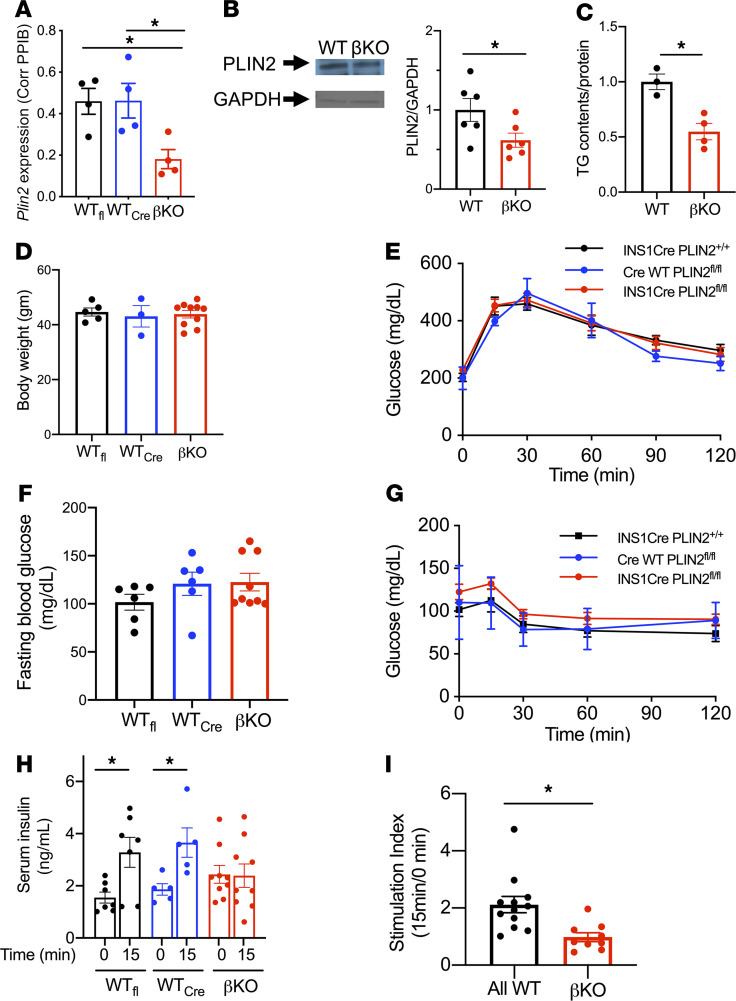
β Cell–specific deletion of PLIN2 impairs insulin secretion from mice on high-fat diets. (**A**) Expression of *Plin2* in β cell–specific PLIN2-KO islets compared with WT littermates determined by qPCR using *Ppib* as an internal control. WT_fl_, INS1Cre PLIN2^+/+^; WT_cre_, CreWT PLIN2^fl/fl^; βKO, INS1Cre PLIN2^fl/fl^. *n* = 4. (**B**) A representative Western blot and densitometry data of PLIN2 corrected for GAPDH. *n* = 6 per group (2WT_fl_ and 4WT_cre_). (**C**) TG contents of islets corrected for protein. Average of WT was taken as 1. *n* = 3 for WT (2 WT_fl_ and 1 WT_cre_) and 4 for βKO. (**D**–**G**) Body weight (BW) (**D**), 1.0 mg/gm BW i.p. GTT (**E**), blood glucose after overnight fasting (**F**), and 0.75 mU/gm BW insulin i.p. ITT on 6-month-old male mice after high-fat diets for 3 months (**G**). (**D** and **E**) *n* = 5 for WT_fl_, 3 for WT_cre_, and 10 for βKO. (**F**) *n* = 6 for WT_fl_, 6 for WT_cre_, and 9 for βKO. (**G**) *n* = 6 for WT_fl_, 2 for WT_cre_, and 9 for βKO. (**H** and **I**) Serum insulin in response to 1.5 mg/gm BW glucose i.p. (**H**) and stimulation index (**I**) determined as the ratio of insulin levels at 15 minutes to levels at 0 minutes for each mouse. *n* = 12 WT (7 for WT_fl_ and 5 for WT_cre_) and 9 for βKO. Data are mean ± SEM for all. *P < 0.05 by Student’s *t* test.

To address whether the increase of PLIN2 seen in β cells under overnutrition is protective or detrimental to β cells, we placed β cell–specific PLIN2-KO mice (βKO) on HFDs, a model that previously resulted in increased TG and PLIN2 levels in islets (12). The efficiency of Ins1(Cre) medicated recombination in β cells (16) was estimated to be above 84% as exon 5 of *Plin2* targeted for excision was reduced to 16% of WT islets in DNA isolated from βKO islets that contain both β and non–β cells (Supplemental Figure 1; supplemental material available online with this article; https://doi.org/10.1172/jci.insight.144341DS1). As expected, islets from βKO mice showed significantly reduced PLIN2 mRNA and protein levels (Figure 1, A and B). The reduction of TG contents, the phenotype typically seen in many models of PLIN2 downregulation (12, 17, 18), was also present in βKO islets, further supporting the reduction of PLIN2 at a functional level (Figure 1C). Body weight (BW) and glucose tolerance did not differ between male WT and βKO mice on regular rodent chow (Supplemental Figure 2, A and B). While 3 months of HF feeding increased BW (Figure 1D versus Supplemental Figure 2A) and blood glucose (Figure 1E versus Supplemental Figure 2B; time 0), PLIN2 deficiency in β cells did not confer better glucose tolerance (Figure 1E) or reduced fasting glucose (Figure 1F). There was also no significant change in insulin tolerance between WT and βKO mice (Figure 1G). However, the acute rise of serum insulin in response to glucose was blunted in βKO mice compared with WT littermates (Figure 1, H and I). Female βKO mice fed HFD also had reduced glucose-stimulated insulin secretion (GSIS) with no differences in BW or glucose tolerance compared with control littermates (Supplemental Figure 2, C–F). Thus, the reduction of PLIN2 in mouse β cells blunts GSIS in vivo, but likely to an extent not severe enough to cause overt hyperglycemia in βKO mice fed HFD.

**Figure 2:**
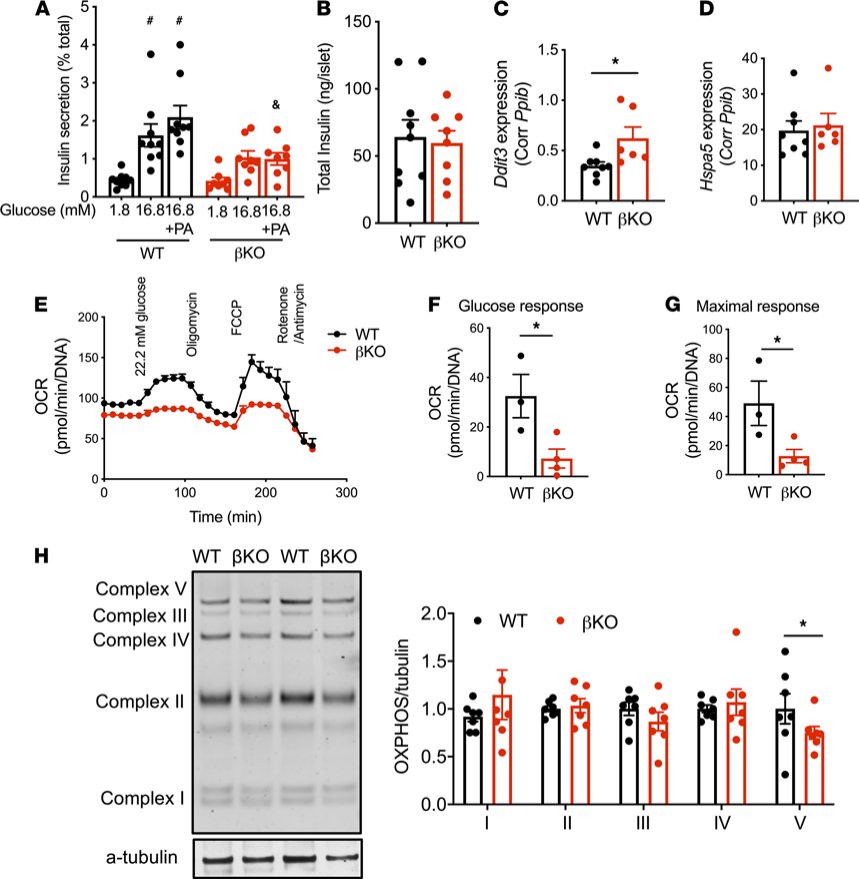
Islets from β cell–specific PLIN2-KO mice on high-fat diets showed impaired insulin secretion and impaired mitochondrial function. (**A**) Insulin secretion measured by static incubation at indicated concentrations of glucose ± 0.5 mM PA for 1 hour corrected for total insulin contents in islets of high-fat diet–fed WT and β cell–specific PLIN2-KO mice (βKO). (**B**) Total insulin contents per islet for **A**. *n* = 9 for WT (3 WT_fl_ and 6 WT_cre_) and 8 for βKO. ^#^*P* < 0.05 versus WT 1.8 mM glucose, ^&^*P* < 0.05 versus WT 16.8 mM glucose + PA by 1-way ANOVA with Sidak’s multiple-comparison test. (**C** and **D**) Expression of *Ddit3* (**C**) and *Hspa5* (**D**) in βKO and WT littermates determined by qPCR using *Ppib* as an internal control. *n* = 8 for WT (5 WT_fl_ and 3 WT_cre_) and 6 for βKO. (**E**) Oxygen consumption rate (OCR) by Seahorse metabolic analyzer in high-fat fed WT (WT_fl_) and βKO islets corrected for DNA. (**F** and **G**) Glucose response (**F**) and maximal respiration (**G**) from **E**. *n* = 3 for WT_fl_ and 4 for βKO. (**H**) Representative Western blot and densitometry data comparing OXPHOS complexes between WT and βKO islets from high-fat diet–fed mice. Data are expressed taking average of OXPHOS/α-tubulin in WT for each complex as 1. *n* = 7 for WT (2WT_fl_ and 5 WT_cre_) and 7 for βKO. Data are mean ± SEM *P < 0.05 by Student’s *t* test.

Insulin secretion was assessed further using islets isolated from WT and βKO mice on HFDs. Here, we focused on male mice that are more susceptible to metabolic effects of HFD than female mice. WT islets appropriately exhibited increase in GSIS, a response augmented by acute exposure to PA (*P* < 0.05 versus 1.8 mM glucose). In contrast, βKO islets did not increase insulin secretion in response to high glucose and PA (not significant versus 1.8 mM glucose; Figure 2A).

The blunted GSIS was not associated with change in total insulin contents (Figure 2B) or β cell area (Supplemental Figure 3, A and B) in βKO islets compared with WT islets. βKO islets showed a mild increase in *Ddit3* without a change in *Hspa5*, indicating PLIN2 deficiency in β cells activates a part of ER stress response under HFD (Figure 2, C and D). The impairment of GSIS in βKO islets was associated with a reduced oxygen consumption rate (OCR) in response to glucose and maximal respiration (Figure 2, E–G). The proton leak was not altered in βKO islets compared with WT islets (Supplemental Figure 3C). βKO islets also showed a significant decrease in mitochondrial complex V protein when compared with WT islets, indicating defects in the mitochondrial respiratory chain (Figure 2H). However, there was no significant difference in mitochondrial DNA between WT and βKO islets (Supplemental Figure 3D).

### PLIN2 deficiency impaired insulin secretion in INS1 cells cultured with and without PA.

**Figure 3:**
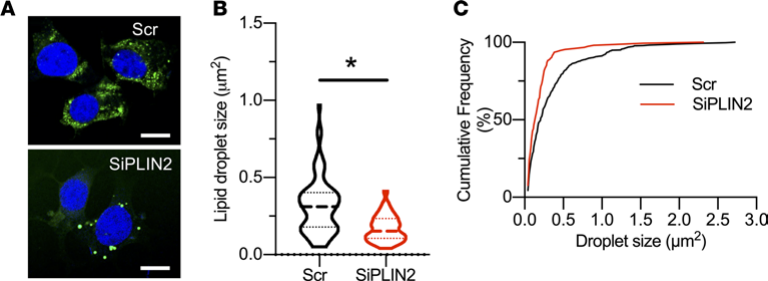
Downregulation of PLIN2 reduces lipid droplets in INS1 cells. (**A**) Representative confocal images of INS1 cells transfected with SiPLIN2 (SiPLIN2) or Scramble control (Scr) stained with Bodipy 493 (green) and DAPI (blue). Scale bar: 10 μm. (**B**) Violin plot for size of an individual lipid droplet (LD) expressed as area. Median and quartiles are indicated. (**C**) Cumulative frequency (%) of **B**. *n* = 69 LDs from 14 cells for Scr and 38 LDs from 13 cells for SiPLIN2. Representative of 3 independent experiments. *P < 0.05 by Student’s *t* test.

**Figure 4:**
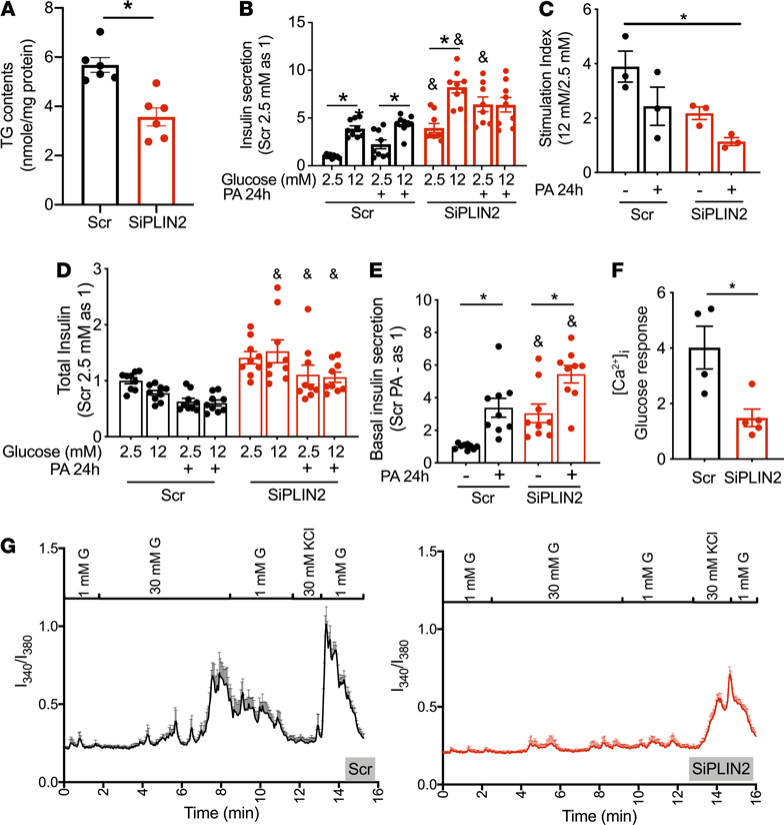
Downregulation of PLIN2 dysregulates insulin secretion and [Ca]_i_ in INS1 cells. (**A**) TG contents corrected for protein contents in Scr and SiPLIN2 transfected INS1 cells. *n* = 6. (**B**–**E**) Insulin secretion at indicated concentrations of glucose in INS1 cells transfected by Scr and SiPLIN2 and preincubated with or without 0.2 mM palmitic acids (PA) for 24 hours. Three experiments each in triplicates. (**B**) Insulin secretion corrected for mg protein was expressed taking the average of Scr-treated at 2.5 mM for each experiment as 1. *n* = 9. (**C**) Stimulation index determined as the ratio of average insulin secretion at 12 mM glucose over 2.5 mM glucose for each experiment. *n* = 3. (**D**) Total insulin contents for **B** were corrected for mg protein and expressed taking the average of Scr-treated at 2.5 mM as 1. *n* = 9. (**E**) Insulin secretion at 2.5 mM glucose per total insulin content (%) was expressed taking the average of Scr-treated at 2.5 mM for each experiment as 1. *n* = 9. (**F** and **G**) Fura-2 [Ca]_i_ transients in INS1 cells in response to 30 mM glucose was measured in 4 cover slips for Scr and 5 for SiPLIN2. (**F**) Glucose response calculated as Fura-2 340/380 as in Methods. (**G**) Representative average tracings. *n* = 8 cells for Scr and 14 cells for SiPLIN2 per cover slide. Supplemental Figure 4D shows tracing of an individual cell. All data are mean ± SEM, except for **B**. *P < 0.05 by Student’s *t* test for (**A** and **F**). One-way ANOVA with Sidak’s post hoc test (**B**, **D**, and **E**) or Bonferroni’s post hoc test (**C**) were performed for multiple comparisons. *P < 0.05 as indicated for **B**, **C**, and **E**; ^&^*P* < 0.05 versus Scr at the same concentrations of glucose and PA.

Data from βKO mouse islets indicate that the reduction of PLIN2 in β cells does not confer protection against HFD. However, the very small size of LDs in mouse islets and the ability of mouse β cells to compensate for HFD feeding might limit the impact of PLIN2 deficiency (10, 19). Thus, we downregulated PLIN2 in INS1 cells, a β cell model that shows similarity to human β cells in the formation of LDs, as shown in Figure 3A and our previous study (20). Also, since PLIN2 is the almost exclusively expressed PLIN isoform in INS1 cells that express little PLIN3, INS1 cells represent an ideal model to test the contribution of PLIN2 (4). Of note, PLIN3 is known to have functional redundancy with PLIN2 (21) and is the second most abundant PLIN in mouse islets (4). After downregulating PLIN2 using siRNA (SiPLIN2) (Supplemental Figure 4, A and B), LDs were reduced both in number (Supplemental Figure 4C) and size (Figure 3, A–C) in INS1 cells, confirming that PLIN2 is a critical determinant of LD mass in INS1 cells. TG contents were reduced after downregulation of PLIN2 in INS1 cells (Figure 4A), as they were in islets from βKO mice (Figure 1C). Then, PLIN2-deficient and control INS1 cells were treated with or without 0.2 mM PA for 24 hours, a dose of PA chosen to cause mild cytotoxicity (22). PA treatment increased insulin secretion at low glucose in INS1 cells treated with scramble (Scr), reducing the stimulation index (SI; Figure 4, B and C). Interestingly, INS1 cells after PLIN2 downregulation showed higher basal insulin secretion and a trend of lower SI in the absence of PA treatment (Figure 4, B and C). Twenty-four–hour treatment with a modest dose of PA (0.2 mM) further increased basal insulin secretion. GSIS was totally lost in SiPLIN2-treated INS1 cells compared with Scr control wherein glucose response was maintained (Figure 4, B and C). Although there was a modest increase in total insulin after the downregulation of PLIN2 (Figure 4D), this did not fully account for the increase in basal insulin secretion. Basal insulin secretion corrected for total insulin was still increased in SiPLIN2-treated INS1 cells compared with control (Figure 4E). Considering that the increase in basal insulin secretion is an early response to nutritional stress in β cell lines, rodent islets, and human islets (23–26), the rise of basal insulin secretion in PLIN2-deficient INS1 cells in the absence of PA implicates that PLIN2 deficiency results in nutritional stress in INS1 cells in regular growth medium. Furthermore, severe blunting of GSIS after 24-hour exposure to PA in SiPLIN2 INS1 cells indicates that PLIN2 is protective against nutritional load in INS1 cells. In agreement with the reduced SI in SiPLIN2 INS1 cells, the rise of [Ca^2+^]_i_ in response to glucose was reduced in SiPLIN2 INS1 cells under regular culture conditions (Figure 4, F and G). Average tracing from 8 Scr-treated cells showed a robust rise of [Ca^2+^]_I_ about 5 minutes after a switch to 30 mM glucose (Figure 4F). While a few cells start to show spikes of [Ca^2+^]_i_ shortly after immersion to 30 mM glucose, an individual tracing of [Ca^2+^]_i_ (Supplemental Figure 4D) indicates that a synchronous spike of [Ca^2+^]_i_ required minutes of exposure to glucose. In response to KCl, Scr-treated INS1 cells showed rapid rise in [Ca^2+^]_i_. In contrast, SiPIN2 INS1 cells showed minimum changes of [Ca^2+^]_i_ in response to 30 mM glucose.

**Figure 5:**
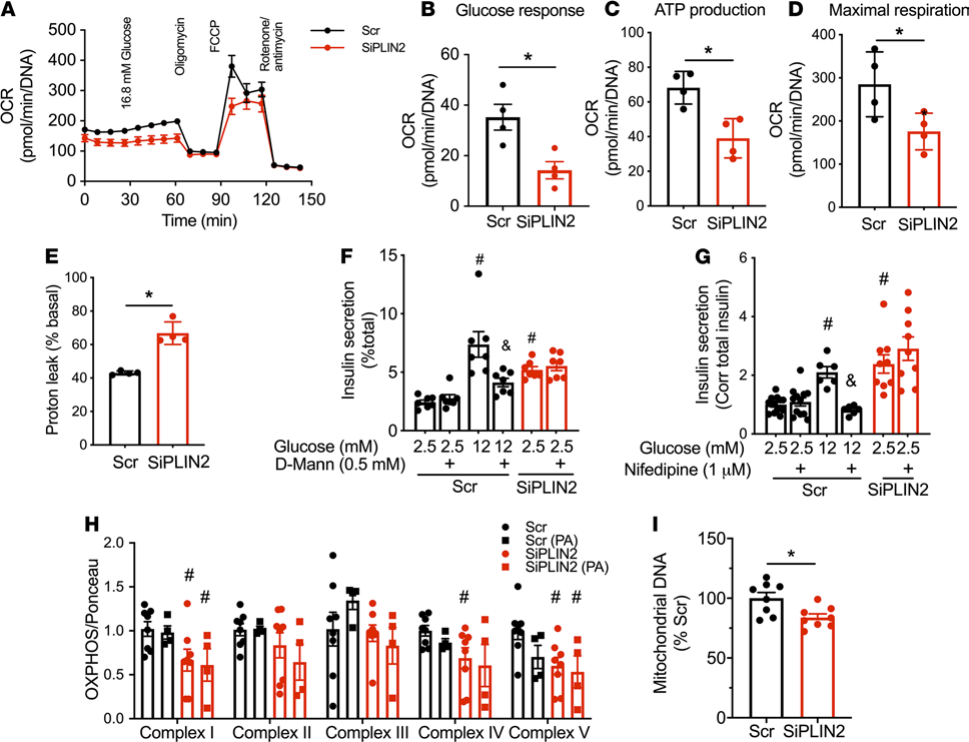
Mitochondrial dysfunction in PLIN2-deficient INS1 cells. (**A–E**) Oxygen consumption rate (OCR) corrected for DNA contents in Scr- and SiPLIN2-treated INS1 cells. (**A**) OCR profile representative of 4 experiments each performed in quadruplicates. (**B**–**E**) Glucose response (**B**), ATP production (**C**), maximal respiration (**D**), and percentage of proton leak (**E**). Each dot is an average value of 1 experiment. *n* = 4. (**F**) Insulin secretion measured with or without 0.5 mM D-Mannoheptulose overnight as in Methods and corrected for total insulin contents. *n* = 7, representative of 3 experiments. (**G**) Insulin secretion measured with or without 1 μM nifedipine for 2 hours as in Methods, corrected for total insulin contents, and expressed taking average of Scr 2.5 mM glucose as 1. *n* =12 for Scr 2.5 mM, 6 for Scr 12 mM, and 9 for SiPLIN2. Representative of 3 experiments. (**F** and **G**) ^#^*P* < 0.05 versus Scr 2.5 mM glucose and ^&^*P* < 0.05 versus Scr 12 mM glucose by 1-way ANOVA with Sidak’s post hoc test. (**H**) Western blot of OXPHOS complex proteins were performed in protein lysate of INS1 cells transfected by Scr and SiPLIN2, treated with or without 0.2 mM palmitic acids (PA) for 24 hours prior to harvest. Densitometry data were corrected for Ponceau S staining and expressed taking average value of Scr without PA as 1 for each complex. *n* = 8 for no PA and 4 for PA-treated INS1 cells. ^#^*P* < 0.05 versus Scr without PA. A representative blot is shown in Supplemental Figure 4E. (**I**) qPCR probed DNA from Scr- and SiPLIN2-treated INS1 cells for mitochondrial DNA (mt-ND6) and nuclear DNA (β actin). Expression of mt-ND6 was corrected for β actin, and the average of value for Scr was taken as 100%. *n* = 8 combined from 3 independent experiments. All data are mean ± SEM. *P < 0.05 by Student’s *t* test.

Similar to islets from βKO on HFD, PLIN2 deficiency in INS1 cells reduced OCR in response to glucose, ATP production, and maximal respiration (Figure 5, A–D). Interestingly, the proton leak was elevated in SiPLIN2-treated INS1 cells that showed increase in basal insulin (Figure 5E). In contrast, βKO islets did not show an increase in basal insulin or an increase in the proton leak (Figure 2A and Supplemental Figure 3C). Recently, the mitochondrial proton leak was proposed to trigger nonsecretagogue insulin secretion under nutritional load through the activation of mitochondrial permeability transition pore (27). Thus, the increase in proton leak in SiPLIN2-treated INS1 cells may be a potential mechanism responsible for the increase in basal insulin secretion. Since accelerated glucose metabolism is another mechanism proposed to increase insulin secretion at low glucose after nutritional stress (23, 25), we tested whether the inhibition of glucokinase by overnight incubation with D-Mannoheptulose reduces basal insulin secretion in SiPLIN2-treated INS1 cells. When 0.5 mM D-Mannoheptulose appropriately reduced insulin secretion at 12 mM glucose in control, basal insulin secretion in SiPLIN2 INS1 cells remained elevated, indicating that enhanced glucose metabolism does not account for high basal insulin secretion in SiPLIN2-treated INS1 cells (Figure 5F). Furthermore, the high level of basal insulin secretion in SiPLIN2 INS1 cells did not depend on Ca^2+^ influx, since nifedipine did not reduce insulin secretion at low glucose in SiPLIN2 INS1 cells (Figure 5G). Mitochondrial complexes I, IV, and V were reduced in SiPLIN2-treated INS1 cultured without chronic PA exposure (Figure 5H and Supplemental Figure 4E). However, the exposure to 0.2 mM PA for 24 hours did not affect the expression of mitochondrial complexes in Scr-treated INS1 cells nor worsen reduction of complexes in siPLIN2-treated INS1 cells compared with PA untreated counterpart (Figure 5H and Supplemental Figure 4E). Mitochondrial DNA was significantly reduced in SiPLIN2 transfected INS1 cells, indicating that mitochondrial mass is reduced after PLIN2 downregulation (Figure 5I). Collectively, downregulation of PLIN2 in INS1 cells impaired mitochondrial function and integrity, sharing the reduction of OCR and OXPHOS complex V with mouse βKO islet on HFD.

### PLIN2 downregulation in INS1 cells increases trafficking of FA to mitochondria.

**Figure 6:**
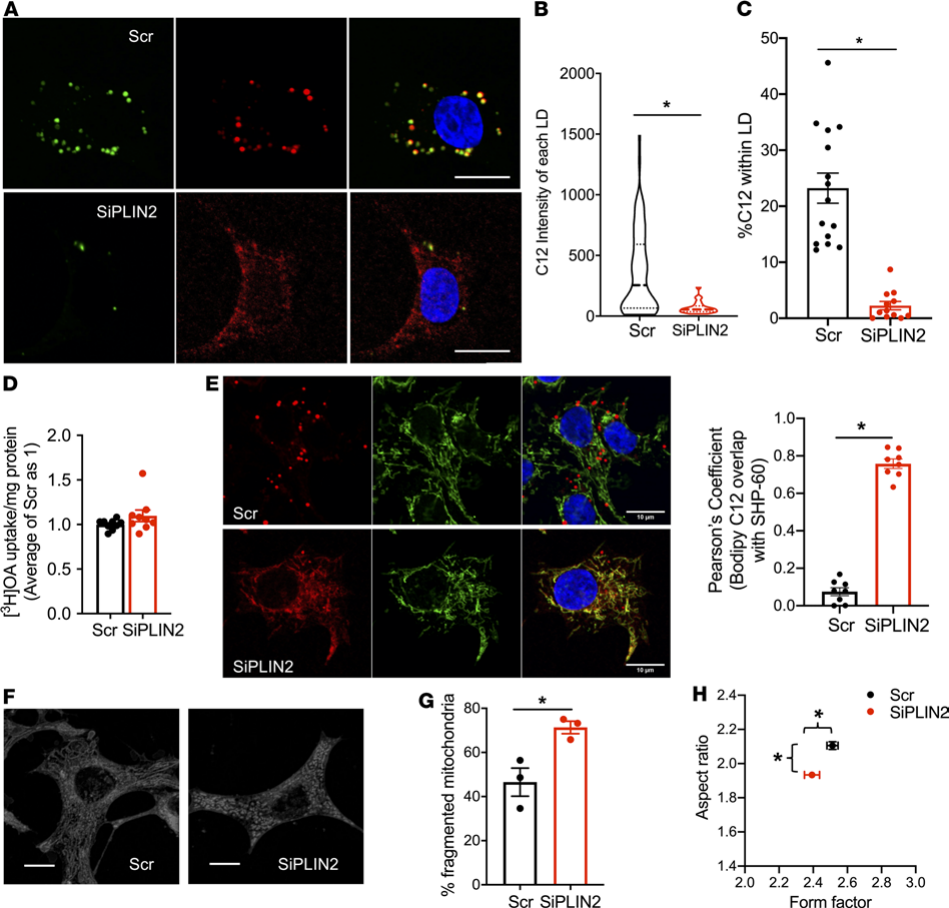
PLIN2 downregulation alters distribution of Bodipy C12 in INS1 cells. (**A**) Representative confocal images of INS1 cells transfected with SiPLIN2 (SiPLIN2) and scramble control (Scr) and metabolically labeled with Bodipy C12 (red) overnight followed by Bodipy 493 (green) and DAPI (blue) staining. Merged pictures on the right. (**B**) Violin plot of integrated intensity of Bodipy C12 signal within each individual lipid droplet (LD) defined by Bodipy 493. Median and quartiles are indicated. *n* = 344 LDs from 15 cells for Scr and 149 LDs from 16 cells for SiPLIN2. (**C**) Proportion of Bodipy C12 signal found in LDs defined by Bodipy 493 in 15 cells for Scr and 14 cells for SiPLIN2 was measured. (**B** and **C**) Representative data from 3 independent experiments. (**D**) [^3^H]OA uptake after overnight incubation of Scr- and SiPLIN2-treated INS1 cells was corrected for protein contents and expressed taking the average value of Scr as 1. *n* = 9. (**E**) Representative confocal images of Scr- and SiPLIN2-treated INS1 cells metabolically labeled with Bodipy C12 (red) overnight followed by immunostaining by HSP60 (green). Merged pictures appear on the right. Pearson’s coefficient for Bodipy C12 and HSP60 was calculated as in Methods. *n* = 8. Representative data from 3 independent experiments. (**F**) Representative MitoTracker Deep Red images of Scr and SiPLIN2 INS1 cells. (**G**) Percentage of fragmented mitochondrial defined as aspect ratio below 2 in 3 independent experiments (exp1–exp3). Number of mitochondria counted for Scr was exp1 = 74, exp2 = 55, exp3 = 81, and for SiPLIN2 was exp1 = 73, exp2 = 41, exp3 = 40. (**H**) An aspect ratio and a form factor for all mitochondria from **H**. *n* = 210 for Scr and 154 for siPLIN2. See Supplemental Figure 4F for violin plot data. Scale bars: 10 μm. Data represent mean ± SEM for all except **B**. *P < 0.05 by Student’s *t* test.

To address why downregulation of PLIN2 affects mitochondria, we investigated how PLIN2 deficiency alters FA distribution within cells by incubating SiPLIN2-treated INS1 cells overnight with Bodipy 558/568 C12 (Bodipy C12), a fluorescent oleic acid (OA) analog that is preferentially incorporated into TG (Figure 6A) (28, 29). In Scr-treated cells, Bodipy C12 was highly incorporated in LD defined by Bodipy 493 (Figure 6, A and B). In contrast, SiPLIN2 caused a significant reduction in the intensity of Bodipy C12 within each LD and a significant reduction in percentage of Bodipy C12 distributed to LDs in each cell (Figure 6, A–C), in agreement with a previous study in MIN6 cells that demonstrated that PLIN2 downregulation reduces [^3^H]OA incorporation into TG measured by thin-layer chromatography (12). Since total OA uptake measured by [^3^H]OA did not differ between Scr- and SiPLIN2-treated cells (Figure 6D), the reduction of LD associated Bodipy C12 implicates the increase of OA distributed outside of LDs in SiPLIN2 cells. Indeed, the high proportion of Bodipy C12 colocalized with a mitochondrial marker, heat shock protein 60 (HSP60), indicating that a larger proportion of FA is targeted to mitochondria in PLIN2-deficient INS1 cells (Figure 6E). We also noted the accelerated fragmentation of mitochondria when SiPLIN2-treated INS1 cells were treated with 0.4 mM OA, which was reported to promote TG formation with minimum cytotoxicity in INS1 cells (Figure 6, F–H) (22). An aspect ratio and a form factor were both decreased in SiPLIN2-treated INS1 cells, indicating that mitochondria are rounder and fragmented — changes also seen in INS1 cells under nutritional stress (Figure 6H and Supplemental Figure 4F) (30, 31).

### PLIN2 downregulation in INS1 cells impacts nutrient metabolism.

**Figure 7:**
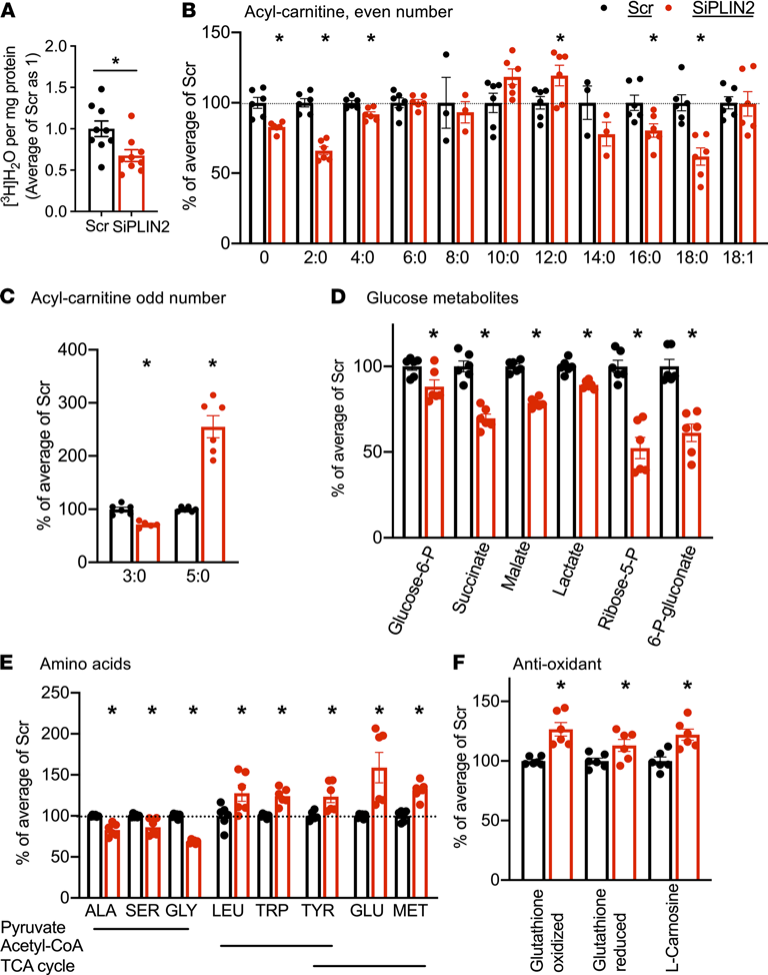
PLIN2 downregulation alters nutrient metabolism in INS1 cells. (**A**) [^3^H]water generated after an overnight incubation with [^3^H]OA in INS1 cells transfected with scramble control (Scr) and SiRNA targeting Plin2 (SiPlin2) corrected for protein contents and expressed taking the average value of Scr as 1. *n* = 9. (**B**–**F**) Relative abundance of metabolites measured by LC-MS/MS in Scr- and SiPLIN2-treated INS1 cells expressed taking the average value of Scr as 100%. *n* = 6 except for C14:0 in **B** that is *n* = 3. Data are mean ± SEM *P < 0.05 by Student’s *t* test.

To address whether the increased targeting of FA to mitochondria alters FA metabolism in SiPLIN2-treated INS1 cells, FA oxidation (FAO) was assessed by measuring the production of [^3^H]water from [^3^H]OA and found to be reduced in SiPLIN2 INS1 cells (Figure 7A). Since the increase in acylcarnitines due to incomplete FAO is proposed to contribute to β cell dysfunction (32–34), we analyzed 81 metabolites that are important for energy homeostasis, including 13 acylcarnitines in SiPLIN2-treated and control INS1 cells under regular culture condition (Supplemental Table 1). Forty-one metabolites, including 8 carnitines, showed statistically significant differences between SiPLIN2 and control INS1 cells, with a clear separation of 2 groups by principal component analysis (PCA; Supplemental Figure 5 and Supplemental Table 2). For acylcarnitines with an even number of acyl carbons, there was overrepresentation of mid-chain C12:0 acylcarnitine, with reduction of both short-chain (C2:0 and C4:0) and long-chain (C16:0 and C18:0) acylcarnitines, consistent with reduced oxidation of mid-chain FA in SiPLIN2-treated INS1 cells (Figure 7B). For odd-chain acylcarnitines, C5:0 was increased but C3:0 was reduced in SiPLIN2-treated INS1 cells (Figure 7C). In addition, the number of glucose metabolites in glycolysis, the TCA cycle, and the pentose phosphate pathway (PPP) was reduced in SiPLIN2-treated INS1 cells (Figure 7D), which could also contribute to impaired GSIS (35). Amino acids primarily metabolized to pyruvate were reduced, but those primarily metabolized to acetyl-CoA and intermediates of the TCA cycle were increased in SiPLIN2-treated INS1 cells (Figure 7E). This likely reflects reduced utilization of amino acids by the TCA cycle, considering that these amino acids were supplied in culture medium. Although the current result is only a snapshot of nutrient metabolism, it is of note that a wide range of metabolites connected with glucose metabolism and important for insulin secretion were altered in PLIN2-deficient INS1 cells. Other significant changes in SiPLIN2-treated INS1 cells included increases in glutathione and carnosine that are known to reduce oxidative stress (Figure 7F) (36, 37). This may compensate for mitochondrial dysfunction and explain the lack of increase in 4-Hydroxynonenal, a marker of oxidative stress, in SiPLIN2-treated INS1 cells (data not shown).

### PLIN2 downregulation does not reduce ER stress in β cell models.

**Figure 8:**
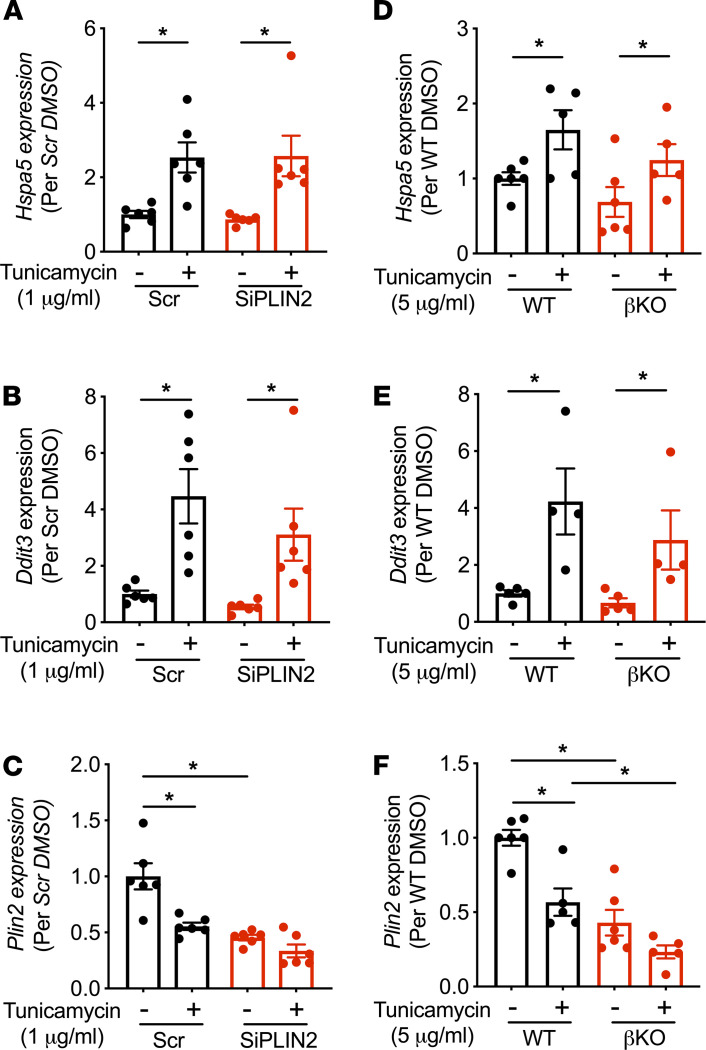
PLIN2 downregulation does not reduce ER stress in INS1 cells and mouse islets. (**A**–**C**) INS1 cells transfected with scramble control (Scr) and SiPLIN2 were treated with DMSO or tunicamycin for 6 hours prior to harvest, and the expression of *Hspa5* (**A**), *Ddit3* (**B**), and *Plin2* (**C**) were measured by qPCR using *Ppib* as internal control. *n* = 6. (**D**–**F**) Expression of *Hspa5* (**D**), *Ddit3* (**E**), and *Plin2* (**F**) in islets of β cell–specific PLIN2 KO (βKO) and WT littermate mice on regular chow cultured with DMSO or tunicamycin for 6 hours prior to harvest. *Ppib* was used as an internal control. *n* = 4–6 for WT_fl_ and βKO. Data are expressed taking the average value of Scr or WT as 1 and mean ± SEM. *P < 0.05 by Student’s *t* test (**A**, **B**, **D**, and **E**) or by 1-way ANOVA with Sidak’s post hoc test (**C** and **F**).

**Figure 9:**
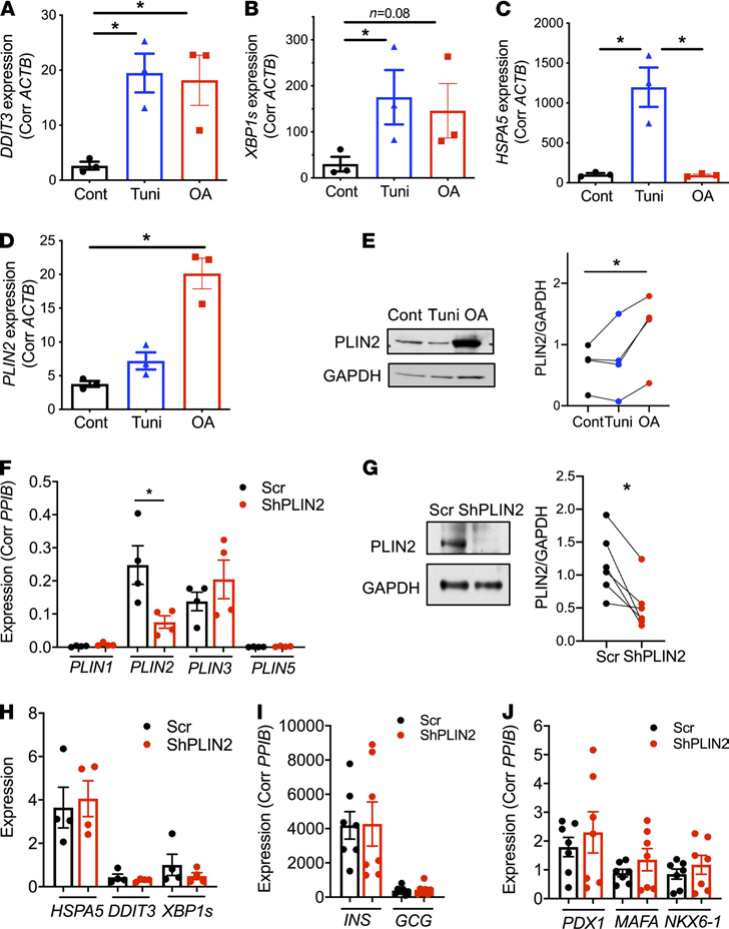
ER stress is not the major driver of PLIN2 expression in human islets. (**A–D**) qPCR compared the expression of *DDIT3* (**A**), *XBP1S* (**B**), *HSPA5* (**C**), and *PLIN2* (**D**) in human islets from nondiabetic donors incubated with 10 μg/mL tunicamycin (tuni) or 0.4 m OA overnight. Data are expressed using *ACTB* as an internal control. *n* = 3 donors. (**E**) A representative blot and densitometry of Western blot comparing the expression of PLIN2 in nondiabetic human islets treated as for **A**–**D**. Data are corrected for GAPDH. Each dot represents a single donor, and data from the same donor are connected by a line. *n* = 4 donors. (**F**) qPCR assessed the expression of PLINs in human pseudoislets treated with lenti-shPLIN2 (shPLIN2) and lenti-shScr (Scr). Expression was corrected using *PPIB* as internal control. (**G**) A representative blot and densitometry of Western blot comparing the expression of PLIN2 in shPLIN2 and Scr human pseudoislets cultured with 0.5 mM OA 16 hours prior to harvest. Data are corrected for GAPDH. *n* = 6 donors. (**H**–**J**) qPCR assessed the expression ER stress markers (**H**), hormones (**I**), and markers of β cell maturation (**J**) in human pseudoislets treated with Scr or shPLIN2. Expression was corrected by *PPIB* as an internal control for all except for *XBP1s*, for which *HPRT1* was used. *n* = 4 for **H** and *n*= 7 for **I** and **J**. All data are mean ± SEM, and each dot represents a single donor. *P < 0.05 by 1-way ANOVA with Sidak’s post hoc test (**A–E**) or by Student’s *t* test (**F** and **G**).

Previously, it was reported that whole body PLIN2-KO mice with an Akita insulin mutation preserve β cell mass compared with Akita mice with intact PLIN2 (13). Thus, we tested whether PLIN2 deficiency in our β cell models reduced ER stress provoked by tunicamycin. While tunicamycin significantly increased *Hspa5* and *Ddit3* expression in control INS1 cells, downregulation of *Plin2* by siRNA did not reduce the expression of *Hspa5* and *Ddit3* significantly in the presence and absence of tunicamycin (Figure 8, A–C). Islets from βKO mice on regular chow were not protected against the tunicamycin-induced rise in *Hspa5* and *Ddit3* expression (Figure 8, D–F). Tunicamycin reduced *Plin2* at mRNA levels in both INS1 cells and mouse islets (Figure 8, C and F), which was somewhat the opposite of a reported increase in *Plin2* level in MIN6 cells and mouse islets after tunicamycin exposure (13). Thus, we assessed how tunicamycin alters PLIN2 expression in human islets. When human islets from nondiabetic donors were incubated with tunicamycin or OA overnight, tunicamycin clearly provoked ER stress, as evidenced by the increase of *HSPA5*, *DDIT3*, and *XBP1s* (Figure 9, A–C). OA showed an increase in *DDIT3* and a trend of increase in *XBP1s*, but no change in *HSPA5* (Figure 9, A–C). However, the increase of PLIN2 at both the mRNA and protein levels was apparent only in OA-treated human islets and not with tunicamycin treatment, indicating that ER stress is not a major driver to increase PLIN2 in human islets (Figure 9, D and E).

### PLIN2 deficiency blunts insulin secretion and reduces OXPHOS complex in human pseudoislets.

**Figure 10:**
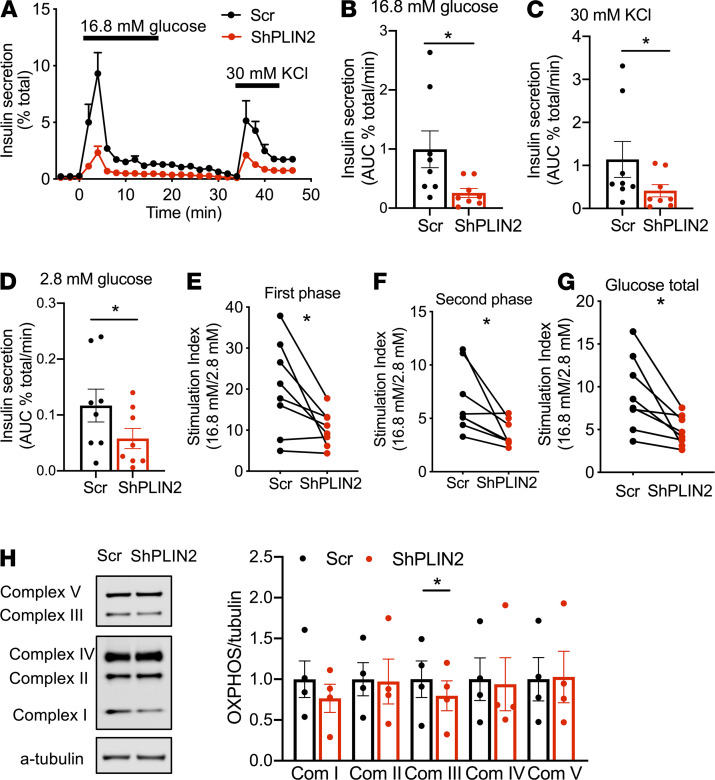
Insulin secretion of human pseudoislets transduced by shPLIN2. (**A**) A representative profile of insulin secretion by perifusion of human pseudoislets transduced by lenti-shPLIN2 (shPLIN2) and lenti-shScr (Scr) in response to 16.8 mM glucose and 30 mM KCl. Data are expressed as percent of total insulin. Mean ± SEM of duplicates from a single donor. (**B**–**D**) AUC of insulin secretion 16.8 mM glucose ramp (**B**), 30 mM KCl (**C**), and 2.8 mM glucose (**D**). Values are expressed per minute as length of ramp differs between treatments. (**E**–**G**) Stimulation index during first phase (**E**), second phase (**F**), and the entire 16.8 mM glucose ramp (**G**) were obtained as in Methods. Each dot represents a single donor, and a line connects data from the same donor. *n* = 8 donors. (**H**) Western blot of OXPHOS complexes in protein lysate of Scr- and shPLIN2-treated human pseudoislets cultured with 0.5 mM oleic acid for 16 hours prior to harvest. Due to difference in intensity of bands, light exposure is shown for complex V and III, and dark exposure is shown for the rest of complexes in the representative blot. Densitometry data were corrected for α-tubulin and expressed taking average value of Scr as 1 for each complex. *n* = 4 donors. All data are mean ± SEM. *P < 0.05 by Student’s *t* test.

Next, we downregulated PLIN2 without changing the expression of other PLINs in human pseudoislets using lenti-shRNA (Figure 9F) (38). The reduction of PLIN2 was also confirmed by Western blot in short hairpin PLN2–treated (shPLIN2-treated) human pseudoislets (Figure 9G). Downregulation of PLIN2 did not change the expression of ER stress markers, *INS* and *GCG,* or maturation markers of β cells, including *PDX1*, *MAFA*, and *NKX6-1*, in human pseudoislets (Figure 9, H–J). However, both glucose-stimulated and KCL-stimulated insulin secretion was significantly reduced to 20% and 30% of control, respectively, in shPLIN2 human pseudoislets cultured in 10% FBS CMRL medium (Figure 10, A–C). There also was moderate reduction of basal insulin secretion to 49% of Scr control in shPLIN2 human pseudoislets (Figure 10D). Since there was a mild but significant increase in total insulin contents in shPLIN2 pseudoislets (1.76- ± 0.20-fold of Scr control, mean ± SEM, *P* < 0.05, *n* = 9), SI was also compared as a parameter independent from insulin contents. Notably, the SIs for first phase (Figure 10E), second phase (Figure 10F), and the entire GSIS (Figure 10G) were all reduced in shPLIN2 human pseudoislets. Since parts of the mitochondrial complexes were reduced in βKO islets from mice on HFD and SiPLIN2-treated INS1 cells, mitochondrial complexes were compared and was found to be reduced for complex III in shPLIN2-treated human pseudoislets (Figure 10H).

## Discussion

Downregulation of PLIN2 negatively affected GSIS in βKO mice fed HFD, in SiPLIN2-treated INS1 cells, and in shPLIN2-treated human pseudoislets, indicating that PLIN2 is indispensable for normal GSIS and that the reduction of PLIN2 accelerates β cell dysfunction under nutritional stress. Blunting of GSIS was associated with reduced OCR in response to glucose in islets from HFD-fed KO mice and in SiPLIN2-treated INS1 cells, pointing to a defect upstream of oxidative phosphorylation. Mitochondrial complex proteins were reduced in βKO islets, in SiPLIN2-treated INS1 cells, and in shPLIN2-treated human pseudoislets, indicating that the integrity of mitochondria is impaired when PLIN2 is downregulated in β cells. The increase in proton leak and fragmentation of mitochondria with OA loading in SiPLIN2-treated INS1 cells further indicates that PLIN2-coated LDs are protective for mitochondria in β cells. PLIN2 deficiency in INS1 cells resulted in reduced levels of FA incorporated into LD and increased trafficking of FA to mitochondria, which might have negatively affected mitochondrial function.

In response to nutritional lipid loading, PLIN2 increased in MIN6 cells, rat islets, mouse islets, and human islets, indicating that PLIN2 is the key PLIN isoform to support expansion of the LD pool during nutrient overload in pancreatic islets (8, 9, 12). Indeed, TG contents were reduced in islet from βKO mice on HFD and in SiPLIN2 INS1 cells. PLIN2 has been proposed to increase LD lipid levels by reducing but not completely blocking lipolysis-mediated by adipose TG lipase (ATGL) (11, 39). Lipolysis corrected for TG accumulation was reduced in HEK293 and MIN6 cells overexpressing PLIN2 (9, 40). Thus, PLIN2 allows expansion of the LD pool in β cells, while maintaining a certain level of lipolysis, which is important for β cells that utilize lipolytic metabolites for insulin secretion (4, 20, 41). In agreement, overexpression of PLIN2 in MIN6 cells increased the TG pool with little effect on GSIS, thereby showing the ability of PLIN2 to expand the inert neutral lipid pool in β cells (9). PLIN2 is stabilized when it interacts with LDs; consequently, PLIN2 enables β cells to quickly adapt to an increase in neutral lipid synthesis by reducing lipolysis (42, 43). The impairment of GSIS in βKO mice on HFD and in SiPLIN2 INS1 cells exposed to PA indicates that upregulation of PLIN2 preserves β cell function when nutritional stress drives expansion of the LD pool. In INS1 cells and in human pseudoislets that possess prominent LDs, impaired insulin secretion was seen even under regular culture conditions after PLIN2 downregulation, further supporting a protective role of LDs for β cell function (20).

Mitochondria can be spatially and functionally associated with LDs through LD formation and FAO (28, 44, 45). Nguyen et al. showed that LD formation buffers FA released by autophagosomal digestion of phospholipids in nutrient-deprived MEF. In that study, LDs prevented mitochondrial dysfunction by limiting exposure of mitochondria to acylcarnitine generated from FA (46). In SiPLIN2-treated INS1 cells, reduction of LD formation increased the distribution of FA to mitochondria, which might have triggered the impairment in complete FAO, leading to an increase in mid-chain acylcarnitines. In β cells, a significant proportion of glucose and FA is directed to TG synthesis within minutes of exposure to glucose, indicating that β cells actively synthesize neutral lipids concurrent with insulin secretion (47, 48). Thus, the formation of PLIN2-coated LDs likely has an important function in protecting mitochondria in β cells, a protection that could become even more important when β cells are exposed to excess FA, as seen in βKO mice fed HFD and in SiPLIN2-treated INS1 cells cultured with PA.

Acylcarnitines, intermediate metabolites of FAO, are known to be increased when FA influx to mitochondria surpasses energy demands and the capacity of the TCA cycle (49). Both long- and medium-chain acylcarnitines were shown to impair mitochondrial function and reduce insulin secretion in mouse islets (33, 34). Recent evidence indicates that acylcarnitines are increased in β cells in T2D and may contribute to β cell dysfunction. Aichler et al. reported the accumulation of stearoyl-carnitine (C18:0) in islets affected by T2D and in mouse models of T2D (33). In our study of SiPLIN2-treated INS1 cells under regular culture conditions, a mid-chain acylcarnitine (C12:0) was increased, while both long-chain acylcarnitines (C16:0, C18:0) and short-chain acylcarnitines (C2:0, C4:0) were reduced. Although the reduction of long-chain acylcarnitines may appear contradictory to the increase in long-chain acylcarnitines reported in T2D islets (33), the discrepancy could be due to the limited availability of carnitine in culture systems. Using mouse islets in culture, Doliba et al. showed that high glucose and FA markedly increase 3-hydroxytetradecaenoyl-carnitine (3-OH-C14), which is known to inhibit oxidative phosphorylation in rat heart mitochondria (32). In Doliba and colleagues’ study, the increase in 3-OH-C14 was also associated with the reduction of C16:0 and C2:0 carnitines in mouse islets cultured with high glucose and PA (32). Alternatively, peroxisomal FAO might have contributed to the reduction of long-chain acylcarnitine in SiPLIN2-treated INS1 cells (50). Also, the study of circulatory acylcarnitines indicated that the increase in mid-chain acylcarnitines may precede the increase in long-chain acylcarnitines during the development of β cell dysfunction (34). Thus, the accumulation of mid-chain acylcarnitine might have mediated mitochondrial dysfunction in PLIN2-deficient β cells.

While the necessity of PLIN2 for GSIS was demonstrated in all 3 models, the rise of basal insulin was seen only in SiPLIN2-treated INS1 cells. The increase in basal insulin is considered to be an early adaptation to nutritional stress and is seen in human islets, mouse islets, and β cell lines exposed to elevated glucose and FA (23–26). Compared with mouse islets and human islets, INS1 cells may be more susceptible to stress from the reduction of PLIN2, since INS1 cells have very low expression of PLIN3, another PLIN with functional redundancy with PLIN2 (21). Alternatively, the short period (72 hours) of intervention to reduce PLIN2 in INS1 cells may account for difference in basal insulin secretion compared with mouse islets (6 months) and human pseudoislets (7 days) that were subjected to longer periods of PLIN2 reduction. It is also important to note that β cell dysfunction in βKO mice on HFD appeared to be mild and insufficient to impair glucose tolerance. Unlike human T2D that is known to be progressive, hyperglycemia in C57BL/6J mice on HFD remains modest as they adapt by increasing β cell mass (19). Morphometry showed that β cell area was similar between βKO and WT littermate mice, implying that PLIN2 deficiency does not affect the adaptive increase in β cell mass. Also, mouse β cells do not make large LDs, as seen in human β cells and INS1 cells (20), which may make the impact of losing PLIN2 less severe. It also needs to be noted that mild phenotype of βKO mice could be due to incomplete knockdown of PLIN2 in our model. The extent of downregulation of PLIN2 in βKO mouse islets determined by quantitative PCR (qPCR) and Western blot (Figure 1, A and B) was less than expected from INS1-Cre efficiency (Supplemental Figure 1). Several possible causes can be considered. Non–β cells may have higher expression of PLIN2 than β cells, reducing the magnitude of decrease in total PLIN2 mRNA and protein in mouse islets. Since PLIN2 protein is known to be stabilized in the presence of lipids posttranslationaly (42, 43), PLIN2 protein levels may remain high even when PLIN2 mRNA is reduced. Due to low expression of PLIN2 (~1/50 of the liver), the ratio of PLIN2 signal over background in mouse islets is low, which may underestimate changes in PLIN2 by Western blot. Nevertheless, our data clearly indicate that the reduction of PLIN2 in β cells does not confer any protection against development of the modest hyperglycemia observed in mice on HFD.

Worsening of β cell function in PLIN2-deficient β cells under HF conditions and PA exposure is in contrast to previous studies of liver and adipose tissue that showed that PLIN2 downregulation reduced adversity associated with nutritional stress. ASO-mediated downregulation of PLIN2 improved insulin sensitivity in C57BL/6J mice fed HFD and in *ob/ob* mice (51). McManaman et al. found that Δ5PLIN2 whole body–KO mice fed HFD were protected against obesity with increased beiging of adipocytes, reduced hepatosteatosis, lower adipose inflammation, and increased mobility (18). One proposed mechanism for the improvement in hepatosteatosis in PLIN2-KO mice is reduced activation of SREBP-1 and -2 in the ER, decreasing TG and cholesterol synthesis. The increase in beiging in Δ5PLIN2 whole body KO was prominent in mice fed high sucrose and correlated with hepatic FGF21 expression, implicating that the loss of hepatic PLIN2 stimulates FGF21 production (52). Given that both hepatocytes and adipocytes have a specialized function in TG metabolism with large LDs and high expression of multiple PLINs, the different consequence of PLIN2 depletion is not necessarily surprising and highlights a cell type–specific role of PLIN2.

It was previously reported that ER stress drives PLIN2 expression in mouse islets and that the reduction of PLIN2 protects β cells from ER stress by increasing autophagy (13). Akita mice with Δ2-3PLIN2 whole body KO were less hyperglycemic than Akita mice with intact PLIN2 (13). However, the reduction of PLIN2 was neutral to tunicamycin induced ER stress and caused a mild increase in *Ddit3* in islets of HFD-fed βKO mice in the current study. It is plausible that the loss of PLIN2 in β cells confers protection under severe ER stress and extreme hyperglycemia, as seen in Akita mice. It is also possible that Δ2-3PLIN2 whole body–KO mice may be less hyperglycemic due to an extra islet effect of PLIN2 deletion, considering that carbohydrates strongly drive FGF21 expression in PLIN2-deficient liver (52). Ultimately, it will be important to determine whether the loss of PLIN2 confers protection to ER homeostasis in human β cells during the development of T2D. Although downregulation of PLIN2 in human pseudoislets was not sufficient to alter markers of ER stress under regular culture condition, it will be important to address whether PLIN2 and LD formation reduce ER stress induced by overnutrition in human islets.

The current study has several limitations. Our data collectively indicate that mitochondria are negatively affected by downregulation of PLIN2 in β cells. However, further study should address whether mitochondrial dysfunction is solely responsible for the impairment in insulin secretion, especially in human islets after PLIN2 downregulation. In human pseudoislets, the impairment of the dynamic secretion of insulin after PLIN2 downregulation was not associated with an increase in markers of ER stress or a reduction in β cells markers. On the other hand, mitochondrial complex III was mildly, but significantly, reduced in shPLIN2 human pseudoislets, indicating that impaired integrity of mitochondria contributes to impaired insulin secretion. However, we are yet to demonstrate the extent of impact of PLIN2 deficiency on multiple aspects of mitochondria in human β cells and to answer whether and how PLIN2 deficiency in human β cells increases FA distribution to mitochondria as seen in INS1 cells. It also should be noted that the lenti-shPLIN2 we utilized is not specific for β cells and can reduce expression of PLIN2 in other human islet cells, as well. While we focused on GSIS in the current study, shPLIN2 human pseudoislets showed defect in KCl-provoked insulin secretion, indicating that there is an additional defect downstream of membrane depolarization, such as the reduction of a readily releasable pool. Further study is required to address a full extent of functional defects after downregulation of PLIN2 in β cells. Since downregulation of PLIN2 was reported not to affect viability of INS1 cells under FA exposure (53), we focused our study of PLIN2 downregulation on β cell function. However, it remains to be determined whether PLIN2 deficiency in human β cells accelerates β cell demise under prolonged nutritional stress in vivo using a model such as transplantation of PLIN2-deficient human pseudoislets to immunodeficient mice on HFD, a model known to increase LD formation in human β cells (10).

In conclusion, PLIN2 is indispensable for normal insulin secretion in HFD-fed mice, in INS1 cells, and in human pseudoislets. A reduction in OXPHOS proteins was noted in all 3 models, indicating that PLIN2 is important to maintain the integrity of mitochondria in β cells. The importance of PLIN2 for mitochondrial health was further demonstrated when the downregulation of PLIN2 in INS1 cells increased FA distribution to mitochondria and caused multiple changes, including reduced OCR, incomplete FAO, alterations in acylcarnitine, reduced glucose metabolism, and susceptibility of mitochondria to fragmentation. Thus, the PLIN2-coated LD is an important organelle to mitigate nutritional stress in β cells.

## Methods

### Animal studies.

Mice with loxP flanking exon 5 of *Plin2* were previously described (18) and crossed with Ins1(Cre) mice (026801, The Jackson Laboratory) on a C57BL/6J background from The Jackson Laboratory (16, 54). Mice were housed 5 per cage in a 12-hour light-dark cycle at 22°C, allowed free access to water, and fed regular rodent chow (7319, Teklad) or HFD (45 kcal% fat, D12451, Research Diets). Mouse islets were isolated by collagenase P (Roche) digestion of the pancreas, followed by Ficoll (GE Healthcare) density centrifugation and cultured overnight in 11 mM glucose RPMI1640 supplemented with 10% FBS, as described (55).

### TG contents.

Mouse islets and INS1 cells were solubilized in RIPA buffer with protease inhibitors, as published (20). Protein concentration of the lysate was measured using RC DC protein assay kit (Bio-Rad). Then, TG was extracted from cell or islet lysate by Folch buffer, as published (12), and quantitated by Infinity Reagents (Thermo Fisher Scientific). TG contents were corrected for protein contents.

### Glucose tolerance test (GTT) and insulin tolerance test (ITT).

GTT was done after 6-hour fasting by loading 1 mg/g BW glucose i.p., and tail blood glucose was measured at time 0, 15, 30, 60, 90, and 120 minutes using a handheld glucometer. We also performed a study using 1.5 mg/g BW glucose i.p. and obtaining blood at time 0 and 15 minutes to measure serum insulin using ELISA (ALPCO). ITT was performed after overnight fasting by 0.75 mU/g BW regular insulin i.p., and glucose was measured at 0, 15, 30, 60, and 120 minutes.

### INS1 cells.

The 823/13 cells (INS1 cells, a gift from Christopher Newgard, Duke University, Durham, North Carolina, USA) were maintained in RPMI1640 supplemented with 10 mM HEPES, 10%FBS, 2 mM L-glutamine, 1 mM sodium pyruvate, 50 μM β-mercaptoethanol, and penicillin + streptomycin (INS1 medium; all from Thermo Fisher Scientific). For downregulation of PLIN2, cells were transfected with 30 nM of siRNA targeting PLIN2 (rn.Ri.Plin2.13.1 from Integrated DNA Technologies) using DharmaFECT1 transfection reagent, as published (20). Nontargeting DsiRNA (Integrated DNA Technologies) was used as negative control. Experiments were performed 72 hours after transfection. Measurement of mitochondrial DNA and Ca imaging was performed as in Supplemental Methods. Metabolomics of INS1 cells transfected with siRNA was performed at the Metabolomic Core Facility of the University of Iowa, as detailed in Supplemental Methods.

### Static incubation.

After a 1-hour preincubation in glucose-free Krebs-Ringer buffer (KRB), INS1 cells, or mouse islets (10 islets/1.5 mL tube) were incubated for 1 hour in KRB containing the indicated concentration of glucose. The insulin secreted and insulin contents were measured with an ultrasensitive mouse Insulin ELISA and STELLUX Chemiluminescent rodent Insulin ELISA, respectively (ALPCO). D-Mannoheptulose (Cayman Chemical) dissolved in DMSO was added overnight and continued through GSIS. Nifedipine (MilliporeSigma) dissolve in DMSO was added 1 hour prior to GSIS.

### mRNA and qPCR.

RNA was isolated from INS1 cells using RNeasy kit (Qiagen) and from islets using TRIzol reagent (Thermo Fisher Scientific), and cDNA was synthesized as published (20). Gene expression was assessed as previously described (20) using ABI TaqMan commercial primers (Applied Biosystems), except for human *XBP1S*, which was assessed using SYBR green master mix (Thermo Fisher Scientific) using primers in Supplemental Table 3.

### Western blot.

Lysates of INS1 cells in RIPA buffer was prepared as for TG contents above. Western blot was performed as described previously (9) using antibodies as listed in Supplemental Table 4. Signal was captured by enhanced chemiluminescence as described (9), except for OXPHOS blots that utilized Odyssey CLx imaging system (Licor).

### OCR.

Respirometry of INS1 cells and mouse islets was performed using an Agilent Seahorse XF24 respirometer (Agilent Technologies), as previously described (20). In brief, the OCR was sequentially measured starting from 5 cycles without glucose, followed by 5 cycles of 22.2 mM glucose, 6 cycles of 5 μM oligomycin, 5 cycles of 1 μM carbonyl cyanide-*4*-(trifluoromethoxy) phenylhydrazone (FCCP), and 6 cycles of 1 μM rotenone + 0.1μM antimycin A (all from MilliporeSigma). Glucose response was defined as the difference between the highest OCR during glucose phase and OCR prior to glucose addition. ATP production was defined as the difference between the highest OCR during the glucose phase and OCR at the end of oligomycin phase. Maximal respiration was defined as the difference between the highest OCR during FCCP phase and OCR at the end of assay. Proton leak was defined as the difference between OCR at the end of oligomycin phase and at the end of assay. All data were normalized to DNA content measured by fluorometric assay using Hoechst 33258 (385 nm excitation and 450 nm emission; MilliporeSigma). Percent protein leak was expressed taking basal as 100% (27).

### Morphometry of LDs in INS1 cells.

INS1 cells were replated onto a cover slip as above and incubated in regular INS1 medium containing 8 μM Bodipy C12 (Molecular Probes) at 37°C at 5% CO_2_ for the last 16 hours of culture. After washing 3 times in PBS, cells were fixed for 15 minutes at 37°C in 4% paraformaldehyde and incubated with 2 μM Bodipy 493/503 (Bodipy 493, Molecular Probes) and 1 μg/mL of DAPI (Thermo Fisher Scientific), as published (9). Images of the cells were captured by a Zeiss LSM710 microscope and analyzed in a blinded fashion by an independent observer. LDs were first identified in each image using the Bodipy 493 channel as follows. The 12-bit images were converted to 8-bit, gaussian blurred with sigma set as 1, and a threshold of 25 was used to separate the LD foreground from both background and diffuse cellular staining. After thresholding, the image was converted to a binary mask, adjacent objects were separated using the watershed function, and the analyze particles function was used to record each droplet as a region of interest (ROI; Supplemental File 1). Once each droplet was identified, the original 12-bit image was opened, and the Bodipy C12 channel was analyzed at each of the ROIs that had been identified using the Bodipy 493 channel. For each ROI, the area and integrated intensity was recorded (Supplemental File 2). In addition, the total integrated intensity across the entire image was recorded. The same data extraction was repeated for every image in the data set. Percent C12 inside LD was calculated by dividing the sum of the integrated intensity of C12 from the ROIs of every droplet in the image by the integrated intensity of the entire image after background subtraction. INS1 cells labeled with Bodipy C12 were also immunostained with mouse anti-HSP60 antibody (66-41-1-lg, Protein Tech) and Alexa Fluor 488 goat anti–mouse IgG (A11017, Invitrogen) both at 1:300. Pearson’s coefficients of Bodipy C12 and Alexa Fluor 488 were calculated on single-plane confocal images captured by a Zeiss LSM710 microscope using the Colocalization function in Bitplane Imaris v 9.6 (Oxford Instruments). The lower threshold was set to 20 for both channels and all images.

### Mitochondrial morphology.

INS1 cells replated onto a cover slip as above were incubated in regular INS1 medium supplemented with 0.4 mM OA (MilliporeSigma) at 37°C at 5% CO_2_ for the last 16 hours. MitoTracker Deep Red (Thermo Fisher Scientific) was added at final 100 nM, and incubation was continued for an additional 30 minutes at 37°C at 5% CO_2_. Then, cells were fixed for 15 minutes at 37°C by adding paraformaldehyde directly at a final concentration of 4%, followed by gentle wash with PBS 3 times. Images of cells were captured by a Zeiss LSM710 microscope and converted to binary images of mitochondrial particles using a custom written NIH ImageJ Marco, as published (56, 57). Automated morphometry of mitochondrial particles was further performed using the NIH ImageJ Marco to obtain aspect ratio (major axis/minor axis) and form factor (perimeter^2^/[4π × area]) (56, 57). Fragmented mitochondria was defined as those with aspect ratio below 2.

### [^3^H]OA oxidation.

Forty-eight hours after transfection, 0.5 μCi/0.5 mL of [^3^H]OA (specific activity 24 mCi/mmole, Perkin Elmer) was added to INS1 cells and cultured overnight at 37°C at 5% CO_2_. The total cellular uptake of [^3^H]OA was measured in cell lysates in RIPA buffer (MilliporeSigma) using liquid scintillation counting and corrected for protein contents. Medium was collected and 10% FA free BSA (MilliporeSigma) was added to remove unincorporated [^3^H]OA. Then, BSA was precipitated by 0.75N TCA (Thermo Fisher Scientific) as published (12), and [^3^H]H_2_O in medium was measured using liquid scintillation counting.

### Induction of ER stress.

Mouse islets were incubated in 10% FBS RPMI1640 medium with 5 μg/mL of tunicamycin (MP Biomedical) for 6 hours. INS1 cells were incubated in INS1 medium with 1 μg/mL of tunicamycin for 6 hours. Human islets were incubated in 1% HSA CMRL1066 medium with 10 μg/mL tunicamycin overnight prior to harvest.

### Human islets.

Human islets from IIDP, Alberta Diabetes Institutes, or Prodo labs (Supplemental Tables 5 and 6) with reported viability and purity above 80% were cultured overnight at 37°C and 5% CO_2_ upon arrival for recovery from shipping (9). Lentivirus carrying scramble (CCTAAGGTTAAGTCGCCCTCG) and shRNA sequence targeting human PLIN2 (CAGAAGCTAGAGCCGCAAATT) obtained from Genetic Perturbation Platform (https://portals.broadinstitute.org/gpp/public) were prepared as previously described (38). Pseudoislets transduced with lentivirus were created in 96-well spheroid plate (Corning) at 2000 cells/well or Aggrewell 400 (StemCell technologies) at 400 cells/well, as described (58), and cultured for 1 week in CMRL 1066 (Thermo Fisher Scientific) supplemented with 10% heat-inactivated FBS, 1% penicillin + streptomycin, and 1% L-glutamate (10% HI-FBS CMRL) at 37°C and 5% CO_2_ before harvesting.

### Perifusion of islets.

BioRep Perifusion System (BioRep Technologies) was used to perifuse human pseudoislets, as published (38). In brief, after 48 minutes in 2.8 mM glucose in KRB, islets were perifused for the indicated time in 16.7 mM glucose or 30 mM KCl in 2.8 mM glucose. Total insulin contents were obtained from islets incubated overnight at 4°C in acidified ethanol. Insulin was measured using STELLUX Chemiluminescent Human Insulin ELISA (ALPCO). Insulin secretion was expressed by taking total insulin contents as 100%. SI for the first phase was determined as the average insulin secretion during the first 6 minutes and SI for the second phase as the average of insulin secretion after 6 minutes until the end of glucose ramp, both divided by average basal insulin secretion.

### Statistics.

Data are presented as mean ± SEM unless otherwise stated in the figure caption. Differences of numeric parameters between 2 groups were assessed with 2-tailed Student’s *t* tests. Pairwise comparison was used for values obtained in human islets from the same donor. Welch correction was applied when variances between 2 groups were significantly different by F test using Prism 8 (GraphPad). Multiple group comparisons used 1-way ANOVA with a post hoc test as indicated. Outlier assessment was performed by ROUT test using Prism 8. *P* < 0.05 was considered significant.

### Study approval.

Experiments using mice were approved by and performed in accordance with the IACUC guidelines of the University of Iowa. The study was determined to be a nonhuman study by the IRB at the University of Iowa.

## Author contributions

YI conceived the study and is responsible for all contents of the manuscript. AM (all aspects), SL (imaging and human islet studies), JP (insulin secretion and mouse studies), MH (human pseudoislet studies), WS and BF (Seahorse metabolic analyzer), GB and BTO (OXPHOS analysis), CK and RS (calcium signaling), S. Strack (mitochondrial morphology), S. Stephens (INS1 cell studies), TK and LJ (mouse studies), FAD and RSA (mouse studies), and JA (morphometric analysis of LD) were responsible for the acquisition and/or analysis of the data. ASG was responsible for establishment of the mouse model and designing of mouse studies. YI, AM, and SL designed research, drafted the manuscript, and critically revised the manuscript for important intellectual content. All authors revised and approved the final version of the manuscript.

## Supplementary Material

Supplemental data

## Version 1 03/30/2021

In-Press Preview

## Version 2 05/10/2021

Electronic publication
