# Supplementary material for: Perilipin 2 downregulation in β cells impairs insulin secretion under nutritional stress and damages mitochondria
Source: JCI Insight. 2021 May 10;6(9):e144341. doi: 10.1172/jci.insight.144341 (PMC8262280; doi:10.1172/jci.insight.144341)
Supplement: Supplemental data [file jciinsight-6-144341-s151.pdf]

Supplementary File 1. ROI identification ImageJ Macro

```
run("8-bit");

run("Arrange Channels...", "new=2");

run("Gaussian Blur...", "sigma=1");

setAutoThreshold("Default dark");

//run("Threshold...");

setThreshold(25, 255);

//setThreshold(25, 255);

run("Convert to Mask");

run("Watershed");

run("Analyze Particles...", "size=0.04-Infinity circularity=0.10-1.00 display exclude clear add");
```

Supplementary File 2. ROI Quantification ImageJ Macro

```
run("Arrange Channels...", "new=12");

roiManager("multi-measure measure_all");
```

Methods for supplementary figures

Isolation of islet DNA and measurement of Plin2 exon 5 by quantitative PCR (qPCR)

Total DNA was isolated from mouse islets by QIAamp DNA minikit (Qiagen). Three primers (P1, P2 and P3 in Supplementary Fig. 1A) surrounding *lox*P sites followed previously published sequences (1). P1 (forward): 5'-AGC AAC CTG ATG GAG ACA CTC AG-3', 86580629-86580607 of NC_000070.7, GRCm39. P2 (reverse): 5'-CAC TGT TCA TGA ACT GCA CCA TC-3', 86580243-86580265 of NC_000070.7, GRCm39. P3 (reverse): 5'-CCG AGA GCA GAG CTT GGT AGA-3', 86578412-86578432 of NC_000070.7, GRCm39. SYBR green real time PCR was performed with P1 and P2 primers using 20 ng of islet DNA as a template to amplify a region between intron prior to an upstream *lox*P site and exon 5 of Plin2. Ndufv1was amplified using 5'-CTT CCC CAC TGG CCT CAA G-3' (forward) and 5'-CCA AAA CCC AGT GAT CCA GC-3' (reverse) primers published by Doliba et al. (2). P1 and P2 product levels were expressed as 2−(delta CT) values using Ndufv1 as an internal control of an unmodified gene. Serial dilution of WT islet DNA was amplified and used to calibrate a linearity of 2−(delta CT) for P1-P2 product.

Morphometric analysis of beta cell area

Morphometric analysis of mouse pancreatic sections was performed as published (3). In brief, whole pancreas was dissected en bloc, fixed in 10% formalin overnight, and paraffin embedded to keep the original shape of pancreas. 7 μm section that contains the maximum footprint was deparaffinized, rehydrated, and immunostained with guinea pig anti-insulin antibody (Ab7842, ABCAM at 1:100) followed by the visualization by Alexa 488 anti-guinea pig antibody at 1:500. Islets were imaged using a Leica DMI6000 B Inverted Microscope by first locating one insulin

positive area and then adjusting fluorescence intensity for best quality images at 10X magnification. GFP settings used for 10X magnification were 300 ms exposure and gain of 16. Next, the microscope was switched to 5X magnification to allow whole pancreas section imaging. Insulin positive sections were then sequentially located in the 5X magnification and imaged using the 10X magnification using preset settings. Once all insulin positive areas were imaged, size of insulin positive area was quantified automatically using ImageJ analysis software.

Mitochondrial DNA

Mitochondrial DNA content was measured by comparing mitochondrial gene (MTeCO1 for mouse islets and Mt-ND6 for INS1 cells) level relative to nuclear gene (Ndufv1 for mouse islets and β actin for INS1 cells) by real-time PCR in total DNA isolated from mouse islets and INS1 cells by QIAamp DNA minikit (Qiagen). Primers were MTeCO1, 5'-TGC TAG CCG CAG GCA TTA C-3' (forward) and 5'-GGG TGC CCA AAG AAT CAG AAC-3' (Reverse); Ndufv1, 5'-CTT CCC CAC TGG CCT CAA G-3' (forward) and 5'-CCA AAA CCC AGT GAT CCA GC-3' (reverse) published by Doliba et al. (2). Mt-ND6, 5′-TTG GGG TTG CGG CTA TTT AT-3′ (forward) and 5′-ATC CCC GCA AAC AAT GAC CA-3′ (reverse); β-actin, 5′-GCT CTA TCA CTG GGC ATT GG-3′ (forward) and 5′-CGC AAC TCT TAA CTC GGA AGA-3′ (reverse) published by Yamazaki et al (4).

Calcium imaging

INS1 cells were re-plated on a cover slip coated with extracellular matrix of HTB9 cells 72h after transfection and calcium imaging was performed as published (5). In brief, cells were

loaded with Fura-2-AM (10 μM) in RPMI1640 at 37 °C for 20 min followed by bathing at 37 °C in the basal KRBH solution containing (in mM): 129 NaCl, 5 $NaHCO_3$, 4.8 KCl, 1.2 KH_2PO_4, 2.5 $CaCl_2$, 2.4 $MgSO_4$, 10 HEPES, 1 glucose, 29 mannitol, 0.1% w/v bovine serum albumin, pH 7.4 with NaOH (300 mOsm/kg) with change to 30 mM glucose or 30 mM KCl at time indicated. Solutions were changed at 2.5 ml/min that is expected to replace the entire bath within 30 seconds. INS1 cells were excited by 340 and 387 nm light alternatively using a DG-4 xenon-arc lamp (Sutter Instruments) and emission signals were recorded at 510 nm every 3 s using CMOS charge-coupled device (CCD) camera (Orca flash 4.0+, Hamamatsu). Images of cells were capture by an Olympus IX73 microscope using a 20 x /0.75 NA objective. The ratio of 340/380 fluorescent signal intensity (*I340/I380*) was used as the measure of $[Ca^{2+}]_i$. Area under the curve (AUC) was obtained from the average tracing for each cover slip. Glucose response was calculated as (AUC during 30 mM glucose- AUC during 1 mM glucose)/AUC during 1 mM glucose.

Metabolomics

INS1 cells transfected with siRNA were washed with PBS. Then, cells were snap frozen on liquid nitrogen, and stored in -80 °C. After overnight lyophilization, dried cells were extracted into an 1 ml/well of methanol/acetonitrile/water (2:2:1 V/V/V) solution containing 1 ng/μl of D4-Succinate, D8-Valine, D4-Citrate, ^{13}C5-Glutamine, ^{13}C5-Glutamate, ^{13}C6-Lysine, ^{13}C5-Methionine, ^{13}C3-Serine, and ^{13}C11-Tryptophan internal standards. The lyophilized cells were scraped for 25 seconds, transferred to a microcentrifuge tube, and flash frozen in liquid nitrogen. Frozen extracts were thawed in a bath sonicator at room temperature (RT) for 10 min, rotated at -20 ºC for 1 h, and centrifuged at 20,000 x g at 4 ºC for 10 min. 400 μl of the supernatants

transferred into new tubes were dried using a Speedvac for 3 h at RT. Dried extracts were reconstituted in 40 µL acetonitrile/water (1:1 v/v), vortexed for 5 min, and centrifuged at 20,000 x g at 4 ºC for 10 min. Finally, 2µL of metabolite extracts were separated using a Millipore SeQuant ZIC-pHILIC (2.1 X 150 mm, 5 µm particle size) column with a ZIC-pHILIC guard column (20 x 2.1 mm) attached to a Thermo Vanquish Flex UHPLC. Mobile phase comprised Buffer A: 20 mM $(NH_4)^2CO_3$, 0.1% NH_4OH and Buffer B: acetonitrile. The chromatographic gradient was run at a flow rate of 0.150 mL/min as follows: 0–20 min—linear gradient from 80 to 20% Buffer B; 20–20.5 min—linear gradient from 20 to 80% Buffer B; and 20.5–28 min—hold at 80% Buffer B (6). Data was acquired using a Thermo Q Exactive mass spectrometer operated in full-scan, polarity-switching mode with a spray voltage set to 3.0 kV, the heated capillary held at 275 °C, and the HESI probe held at 350 °C. The sheath gas flow was set to 40 units, the auxiliary gas flow was set to 15 units, and the sweep gas flow was set to 1 unit. MS data was acquired in a range of m/z 70–1,000, with the resolution set at 70,000, the AGC target at 10e6, and the maximum injection time at 200 ms. Acquired LC-MS data were processed by Thermo Scientific TraceFinder 4.1 software, and metabolites were identified based on the University of Iowa Metabolomics Core facility in-house, physical standard-generated library. NOREVA was used for signal drift correction (7). Data per sample were then normalized to total ion signal per sample (Supplementary Table 1) and MetaboAnalyst 4.0 was used for further statistical processing and visualization (8).

Principal Component Analysis

Principal Component Analysis (PCA) was performed using MetaboAnalyst 4.0 software for metabolomic data (Supplementary Table 1) obtained from 2 independent experiments of INS1

cells treated with Scr control and SiPLIN2 as in Methods, each performed in triplicate. A 2D scores plot comparing the top 2 PC calculated is shown in Supplementary Figure 4.

Supplementary Figure

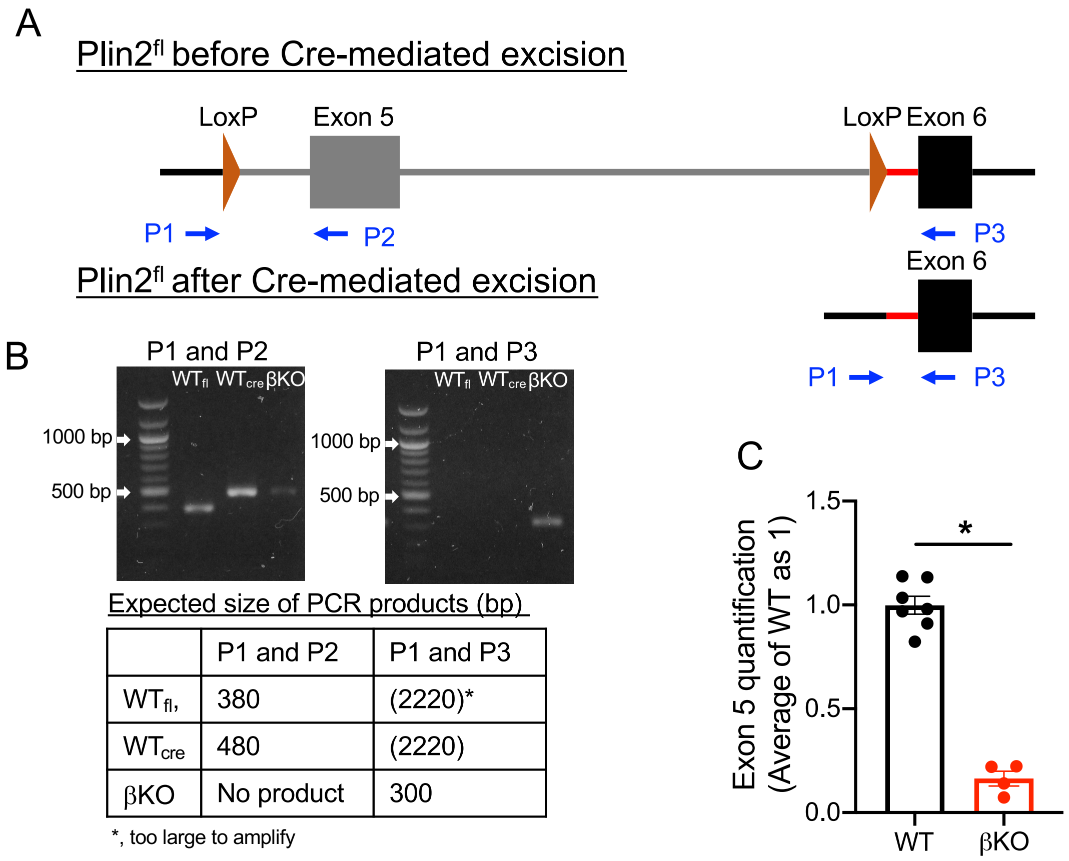

	P1 and P2	P1 and P3
WT$_{fl}$,	380	(2220)*
WT$_{cre}$	480	(2220)
βKO	No product	300

Figure 1. Efficiency of Cre-mediated recombination of Plin2 in Ins1Cre PLIN2$^{fl/fl}$ mice
(A) *Lox*P sites flanks exon 5 of the Plin2 locus in PLIN2$^{fl/fl}$ mice used in the study (1). The location of three primers in Supplementary methods are shown in blue. (B) Expected size of PCR products and representative gel images of PCR products for islet DNA isolated from WT$_{fl}$; INS1Cre PLIN2$^{+/+}$, WT$_{cre}$; CreWT PLIN2$^{fl/fl}$, and βKO; INS1Cre PLIN2$^{fl/f}$ mice. 100 bp DNA ladder (New England BioLabs) is included. (C) qPCR compared abundance of P1-P2 products using 20 ng of islet DNA as a template in 7 WT (2 WT$_{fl}$ and 5 WT$_{cre}$) and 4 βKO mice. Data are mean ± s.e.m. *; $p<0.05$ vs WT$_{fl}$ and WT$_{cre}$ by Student’s t test.

Figure 2. Metabolic profiling of beta cell specific PLIN2 deficient mice.

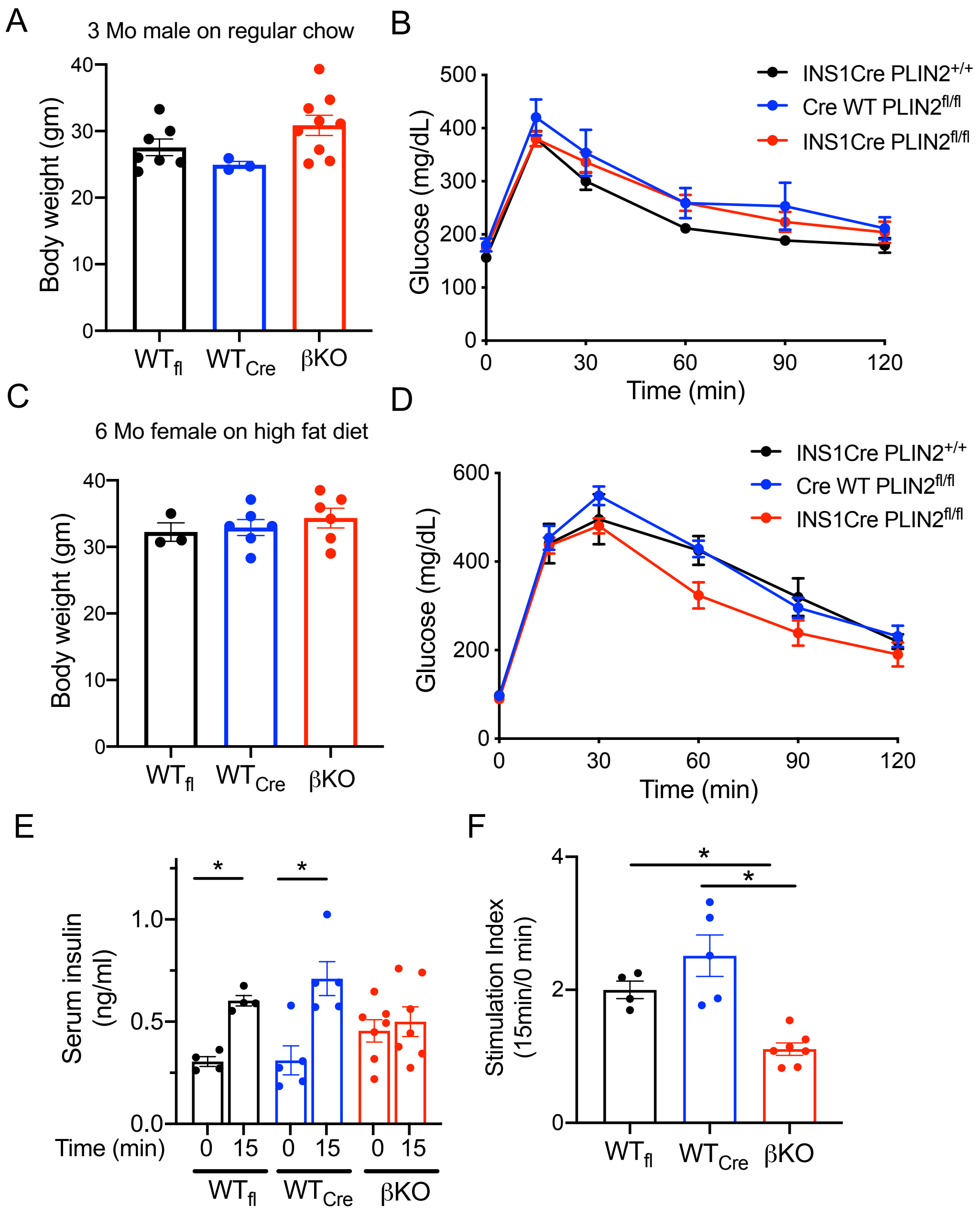

(A) body weight (BW) and (B) 1.5 mg/g BW glucose GTT was done on 3-month old male mice on regular rodent chow after 6 h fasting. WT_{fl}; INS1Cre $PLIN2^{+/+}$, WT_{cre}; CreWT $PLIN2^{fl/fl}$, βKO; INS1Cre $PLIN2^{fl/fl}$. n= 7 for WT_{fl}, 3 for WT_{cre}, and 9 for βKO. (C) BW and (D) 1.0 mg/g BW glucose GTT was done on 6-month old female mice on high fat diet chow for 6 weeks after 6 hour fasting. WT_{fl}; INS1Cre $PLIN2^{+/+}$, WT_{cre}; CreWT $PLIN2^{fl/fl}$, βKO; INS1Cre $PLIN2^{fl/fl}$. n= 3 for WT_{fl}, 6 for WT_{cre}, and 6 for βKO. (E) Serum insulin in response to 1.5 mg/g BW i.p. glucose GTT and (F) stimulation index determined as insulin level at 15 min over 0 min for each female mouse. n= 4 for WT_{fl}, 5 for WT_{cre}, and 7 for βKO. All data are mean ± s.e.m.

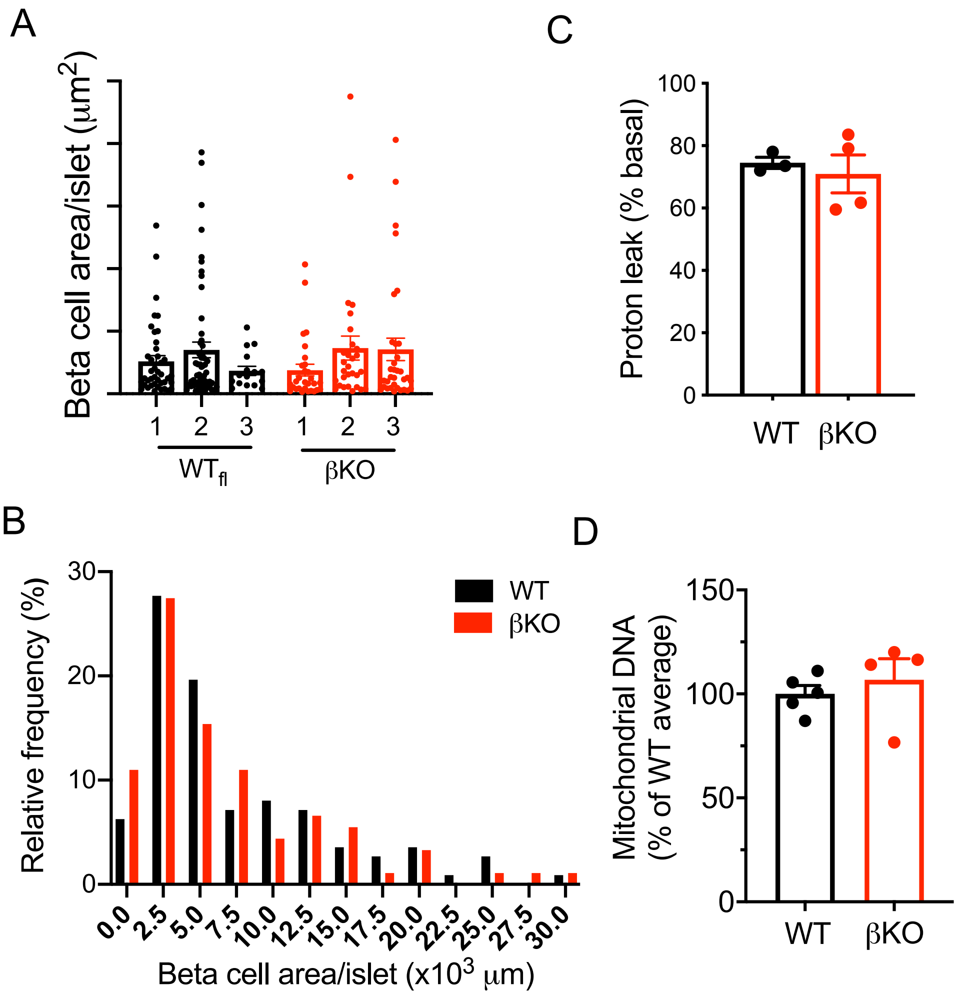

Figure 3. Islet data from beta cell specific PLIN2 deficient mice.

(A-B) Beta cell area per islet was defined by measuring insulin positive area as in Supplementary methods in pancreatic section from 3 WT (WT_{fl};INS1Cre PLIN2$^{+/+}$) and 3 βKO (INS1Cre PLIN2$^{fl/fl}$) mice placed on HFD. (A) Mean ± s.e.m. of islet size for each mouse. n= 41 for WT1, 55 for WT2, 16 for WT3, 29 for KO1, 29 for KO2, and 33 for KO3. (B) Distribution of islet size defined as beta cell area. Data combines all islets from 3WT and 3 βKO from (A). n=112 for WT and 91 for βKO. (C) Proton leak expressed as % basal oxygen consumption rate (OCR) determined by Seahorse Metabolic analyzer in mouse islets from high fat fed WT (WT_{fl}) and βKO islets corrected for DNA. n=4 for WT_{fl} and 3 for βKO. (D) qPCR probed DNA from WT and βKO islets for expression levels of mitochondrial DNA (MTeCO1) and nuclear DNA (Ndufv1). Expression of MTeCO1 was corrected for Ndufv1 and the average of value for WT was taken as 100%. Data are mean ± s.e.m. n=5 for WT (1WT_{fl} and 4WT_{cre}) and 4 for βKO.

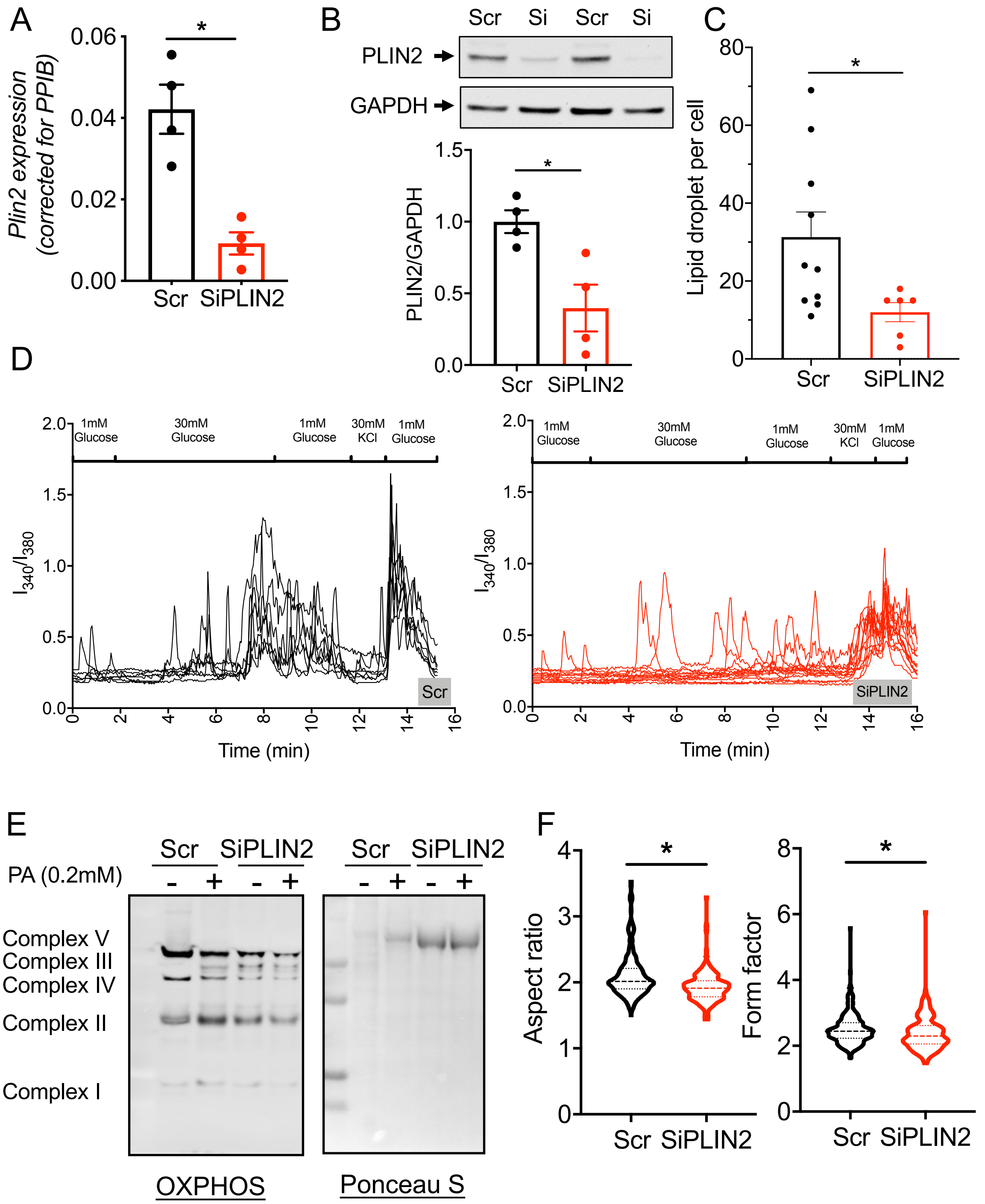

Figure 4. Down-regulation of PLIN2 in INS1 cells

(A) Expression of *Plin2* in INS1 cells transfected with scramble control siRNA (Scr) or siRNA targeting Plin2 (SiPlin2) determined by qPCR using *Ppib* as an internal control. n= 4. (B) Western blot compared the expression of PLIN2 in SiPLIN2 and Scr treated INS1 cells. A representative Western blot and densitometry data corrected for GAPDH. n=4. (C) Number of lipid droplets (LDs) per cell in 12 cells treated by Scr and 6 cells treated by SiPLIN2 were

measured. Representative of 3 independent experiments. (D) Tracing of Fura-2 Ca^{2+} transients in INS1 cells transfected with Scr or SiPlin2 on a cover slip in response to 30 mM glucose (basal 1 mM glucose) from Fig. 4f plotted to show an individual cell. n= 8 cells for Scr and 14 cells for SiPLIN2 per cover slide. (E) Representative Western blot for Figure 5h that probed OXPHOS complex proteins in protein lysate of INS1 cells transfected by Scr and SiPLIN2 treated with or without 0.2 mM palmitic acids (PA) for 24 h prior to harvest. (F) Distribution of aspect ratio and form factor shown as violin plots for all mitochondria counted in three independent experiments n= 210 for Scr and 154 for siPLIN2. Medium and quantiles are shown. For all others, data are mean ± s.e.m. *; $p<0.05$ by student's t test.

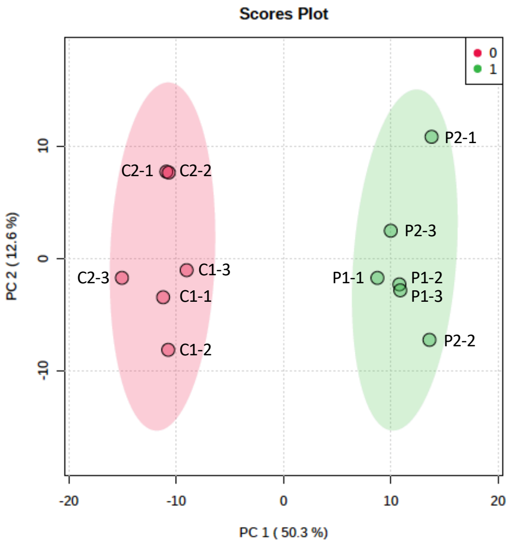

Figure 5. Principal component analysis compared metabolites between Scr and SiPLIN2 treated INS1 cells

% variance explained by principal component (PC)1 is shown in x axis and PC2 in y axis. Experiments were repeated twice (Exp1 and 2) each in triplicates and the results were combined making total 6 samples per treatment. Scr control (C1, C2) values were shaded red and siPLIN2 (P1, P2) values in green. See Supplementary Table 1 for raw data.

Supplementary Table 1: List of metabolites measured

Name	KEGG[a]	HMDB[b]	EXP 1						EXP2					
			Scr			SiPLIN2			Scr			SiPLIN2		
			1	2	3	1	2	3	1	2	3	1	2	3
2-Deoxy-guanosine 5-triphosphate sodium salt hydrate	C00286	HMDB 0001440	9.39	7.75	9.67	8.94	8.48	8.26	11.77	11.05	8.83	13.53	8.20	12.60
4-Imidazoleacrylic acid	C00785	HMDB 0000301	10.80	8.68	7.02	7.44	7.87	5.30	9.31	8.89	21.25	4.57	7.36	19.17
5-Deoxy.5. methylthio. adenosine	C00170	HMDB 0001173	8.50	7.91	8.75	11.25	9.63	9.50	9.92	9.60	10.48	10.46	10.62	10.22
6-phospho-gluconate	C00345	HMDB 0001316	11.10	9.05	9.33	7.25	6.35	7.13	13.33	13.07	15.97	9.02	6.00	7.04
Adenine	C00147	HMDB 0000034	8.24	8.33	8.17	11.63	9.24	9.17	10.18	10.21	10.68	11.13	10.58	11.34
Adenosine	C00212	HMDB 0000050	10.10	9.91	9.30	7.73	7.91	7.26	14.44	9.83	10.75	9.92	8.74	8.53
ADP	C00008	HMDB 0001341	9.01	5.35	8.49	7.97	5.82	7.24	9.29	12.18	7.84	14.07	12.81	11.40
Alanine	C00041	HMDB 0000161	8.89	8.94	9.10	7.99	8.35	7.70	11.80	11.70	12.04	9.12	9.36	8.66
Allantoin	C01551	HMDB 0000462	9.21	9.25	8.52	8.76	8.62	8.38	10.37	10.91	10.85	10.63	11.07	9.66
AMP	C00020	HMDB 0000045	9.33	9.14	9.39	8.05	8.54	8.31	11.28	11.33	9.03	11.41	10.25	11.51
ATP	C00002	HMDB 0000538	9.39	7.75	9.67	8.94	8.48	8.26	11.77	11.05	8.83	13.53	8.20	12.60
B.Nicotinamide. mononucleotide	C00455	HMDB 0000229	4.04	5.18	5.91	8.72	11.08	10.40	8.19	12.15	9.93	10.79	12.08	9.20
Butyryl.L.carnitine	C02862	HMDB 0002013	8.96	9.65	8.85	8.92	8.27	8.84	11.04	10.99	10.37	9.89	9.49	9.36
Citrulline	C00327	HMDB 0000904	7.64	8.37	8.38	8.84	9.82	9.18	10.00	9.81	10.03	11.02	11.33	11.22
CMP	C00055	HMDB 0000095	11.78	10.46	15.07	7.60	9.62	7.81	12.01	12.27	11.77	9.68	9.07	7.94
Creatine	C00300	HMDB 0000064	9.12	9.30	8.86	8.34	8.79	7.39	9.86	11.05	10.80	9.72	10.47	9.52
Cytidine	C00475	HMDB 0000089	9.93	10.04	9.82	7.98	8.69	7.80	12.82	12.31	13.67	7.91	7.45	7.75
Cytidine.5.diphosphocholine.sodium.salt. dihydrate	C00307	HMDB 0001413	7.98	9.19	9.26	7.20	9.62	8.01	10.09	9.90	9.43	10.45	12.24	10.76
Decanoyl.L.carnitine	C03299	HMDB 0062631	9.04	13.38	13.48	12.21	13.41	12.81	7.59	9.48	10.49	12.61	11.39	11.79
D.Gluconic.acid.solution	C00257	HMDB 0000625	8.66	8.61	8.50	9.14	8.75	9.05	11.31	11.79	11.49	10.16	10.38	9.98
D.Glucose.6.phosphate.potassium.salt	C00092	HMDB 0001401	10.43	9.89	9.78	7.46	7.68	7.43	11.81	11.89	10.81	9.57	9.16	9.60
DL. Homocysteine	C05330	HMDB 0000742	9.32	7.65	9.93	7.97	8.54	8.69	11.01	11.41	9.01	10.58	9.52	11.27
DL. lauroylcarnitine	None	HMDB 0002250	12.24	12.13	9.48	10.86	11.13	14.98	8.20	9.20	9.98	10.96	12.38	12.11
DL.Leucine	C00123	HMDB 0000687	5.18	7.29	7.91	9.20	10.42	10.65	11.24	10.12	10.89	11.81	11.89	10.75
D.Ornithine.monohydrochloride	C00515	HMDB 0003374	9.10	8.48	8.40	8.41	8.78	8.78	10.45	10.59	9.66	10.22	10.08	9.95
D.Tryptophan	C00525	HMDB 0013609	8.09	7.90	7.55	10.74	9.95	10.32	9.89	9.67	9.74	11.72	11.63	10.68
FAD	C00016	HMDB 0001248	9.47	8.73	9.48	8.35	8.66	8.45	10.49	10.41	8.82	11.47	9.60	10.52

GDP	C00035	HMDB 0001201	8.72	8.96	8.95	8.62	9.65	7.87	11.12	13.61	9.10	14.61	9.51	12.01
Glutamate	C00025	HMDB 0000148	8.54	8.90	8.49	8.95	9.32	8.85	10.84	11.33	11.14	9.49	9.49	9.24
Glutamine	C00064	HMDB 0000641	5.99	5.94	5.73	12.16	11.65	11.53	9.74	9.09	9.69	11.33	11.47	10.78
Glutathione. oxidized.	C00127	HMDB 0003337	7.53	8.05	7.56	8.81	9.11	8.80	8.86	8.27	8.43	12.11	10.79	12.31
Glutathione. reduced.	C00051	HMDB 0000125	9.69	8.95	8.28	9.89	8.61	9.14	8.94	9.26	9.86	11.42	11.86	11.35
Glycerol	C00116	HMDB 0000131	10.60	8.38	9.01	9.47	7.53	7.57	8.20	7.96	7.51	7.07	20.30	6.68
Glycine	C00037	HMDB 0000123	9.02	9.33	9.21	8.35	8.93	8.94	11.92	11.33	11.34	8.93	8.74	9.00
GMP	C00144	HMDB 0001397	9.77	9.29	9.76	7.61	9.14	8.55	8.80	10.43	9.23	12.27	11.32	11.45
GTP	C00044	HMDB 0001273	8.34	7.04	8.69	9.27	7.92	7.81	14.29	16.82	9.42	18.03	6.87	12.62
Guanosine	C00387	HMDB 0000133	9.65	9.94	9.01	7.99	8.51	7.90	10.27	10.52	9.85	10.22	9.80	9.75
Hexanoyl. L.carnitine	None	HMDB 0000756	8.98	8.80	8.38	8.69	8.61	8.13	8.93	11.41	11.00	10.98	10.52	11.00
Hypotaurine	C00519	HMDB 0000965	8.12	8.11	7.76	9.23	9.53	9.01	10.42	10.40	10.84	10.07	10.62	10.48
Hypoxanthine	C00262	HMDB 0000157	7.42	6.92	6.26	9.69	10.85	10.91	5.60	5.71	5.93	14.84	16.56	14.84
IMP	C00130	HMDB 0000175	8.04	9.50	9.21	9.49	11.26	10.68	7.20	7.50	6.82	14.44	13.78	12.74
Inosine	C00294	HMDB 0000195	8.93	9.76	8.96	9.80	10.67	11.96	8.79	9.11	10.03	10.25	11.87	10.61
Isoleucine	C00407	HMDB 0000172	9.74	9.58	10.79	9.46	8.81	9.78	10.10	9.88	10.66	11.11	11.26	10.70
L. Acetylcarnitine	C02571	HMDB 0000201	10.03	11.05	9.27	7.59	6.90	7.39	11.73	13.56	13.07	7.81	7.63	7.52
Lactate	C00186	HMDB 0000190	9.77	8.65	9.12	8.20	8.11	8.50	10.88	10.74	11.11	9.96	9.52	9.44
L.Asparagine	C00152	HMDB 0000168	8.96	8.82	8.84	9.00	9.15	8.49	11.02	10.63	10.87	9.73	10.18	9.73
L.Aspartic.acid	C00049	HMDB 0000191	7.61	7.61	7.43	10.42	9.95	9.89	10.50	10.05	10.49	10.58	10.47	10.17
L.Carnitine	C00318	HMDB 0000062	10.00	10.61	8.55	8.41	7.41	8.05	9.98	12.28	10.99	9.26	9.44	9.14
L.Carnosine	C00386	HMDB 0000033	7.83	7.06	8.88	9.76	11.14	10.26	10.24	9.36	9.35	10.60	11.20	10.91
L.Cystathionine	C02291	HMDB 0000099	8.65	9.80	8.89	9.70	9.80	8.86	9.67	12.03	10.10	9.88	11.34	10.41
L.Dihydroorotic. acid	C00337	HMDB 0003349	8.21	5.60	6.18	8.66	6.32	8.30	7.41	10.81	10.59	10.64	12.13	10.53
L.Homoserine	C00263	HMDB 0000719	7.87	8.06	8.11	8.51	9.26	8.22	11.05	9.46	10.57	10.30	10.36	9.03
L.Kynurenine	C00328	HMDB 0000684	8.24	8.19	9.08	8.25	10.36	13.25	11.96	11.07	11.54	11.77	12.05	10.06
L.Malic.acid	C00149	HMDB 0000156	9.76	9.78	9.11	7.92	7.50	7.71	11.27	12.04	11.39	8.70	9.00	8.89
L.Phenylalanine	C00079	HMDB 0000159	7.37	7.43	7.54	10.11	10.69	10.59	10.76	9.53	9.68	11.22	11.52	10.58
L.Tartaric.acid	C00898	HMDB 00956	7.57	8.43	12.59	6.18	7.39	10.93	8.30	11.33	13.71	9.53	6.27	11.19
L.Tyrosine	C00082	HMDB 0000158	7.06	7.18	7.56	9.56	10.40	10.43	11.02	10.03	9.56	10.79	11.10	11.02
L.Valine	C00183	HMDB 0000883	8.90	8.23	8.23	9.20	9.45	8.97	11.29	10.46	11.62	10.69	10.88	10.52
Malonic.acid	C00383	HMDB 0000691	6.09	7.13	10.20	6.06	6.74	10.92	10.38	10.16	10.48	10.37	11.02	12.57
Methionine	C00073	HMDB 0000696	7.76	7.44	6.80	9.34	9.79	10.68	9.91	8.52	9.72	10.67	12.70	12.39
N.Acetyl. L.aspartic.acid	C01042	HMDB 0000812	7.23	8.29	6.82	9.66	10.16	10.24	8.05	8.51	7.65	13.79	12.30	12.72
N.acetyl. neuraminic.acid	C19910	HMDB 0000230	11.73	10.84	10.64	6.05	6.51	6.66	13.94	14.08	13.43	7.48	7.17	7.53

NAD	C00003	HMDB0000902	9.19	9.62	9.08	8.81	9.09	8.37	12.04	11.55	10.76	10.42	9.90	9.66
NADH	C00004	HMDB0001487	9.53	7.42	10.91	11.33	7.84	10.49	14.25	9.38	8.89	12.78	8.59	12.71
NADP	C00006	HMDB0000217	9.49	7.85	9.75	7.62	7.90	8.29	9.34	11.61	8.67	12.76	9.76	10.90
NADPH	C00005	HMDB00221	8.57	7.02	8.26	9.56	9.25	8.42	13.32	8.26	8.95	12.25	8.73	12.44
N.alpha.Acetyl.L. asparagine	None	HMDB0006028	7.63	8.94	8.74	10.59	10.43	8.81	10.45	10.29	10.93	10.32	11.29	9.64
Nicotinamide	C00153	HMDB0001406	9.03	8.90	9.10	9.11	7.49	8.13	10.80	10.21	11.55	10.38	11.92	10.58
Nicotinamide. hypoxanthine. dinucleotide. sodium.salt	C04423	None	7.23	7.15	8.93	9.87	12.45	10.45	9.65	8.97	6.40	10.62	13.38	11.65
Nicotinate	C00253	HMDB0001488	9.00	8.22	10.15	8.06	8.69	8.64	11.56	9.09	10.68	10.05	10.85	10.14
N.Methyl. D.aspartic.acid	C12269	HMDB0002393	8.07	8.32	8.11	9.68	10.17	9.56	10.74	10.87	10.53	9.72	9.85	9.53
Octanoyl-L-carnitine	C02838	HMDB0000791	9.41	7.43	13.65	9.62	8.08	10.73	nd	nd	nd	nd	nd	nd
Oleoyl. L.carnitine		HMDB0005065	11.85	12.80	10.14	9.95	8.89	9.69	8.97	9.26	11.16	9.75	12.17	12.35
O.Phosphorylethanolamine	C00346	HMDB0000224	9.42	8.16	8.18	8.68	8.71	8.61	8.31	8.48	8.46	12.07	12.22	12.58
Palmitoyl. L.carnitine	C02990	HMDB0000222	9.50	11.24	8.37	7.91	6.09	7.37	10.22	10.28	13.09	8.52	10.15	10.63
Phosphocreatine	C02305	HMDB0001511	8.81	8.53	8.93	8.38	8.96	8.26	11.00	10.24	10.11	10.12	11.30	10.07
Proline	C00148	HMDB0000162	8.04	8.26	8.37	8.77	9.28	8.37	11.52	11.23	10.80	9.90	10.29	9.95
Propionyl. L.carnitine	C03017	HMDB0062514	10.06	11.67	9.16	8.11	7.29	7.65	10.89	12.51	12.46	0.07	8.26	7.64
Pyridoxine	C00314	HMDB0000239	10.36	11.26	8.34	7.49	6.31	8.30	12.21	10.04	10.72	10.24	10.68	9.28
Ribose.5. phosphate	C00117	HMDB0001548	12.19	10.82	9.73	7.70	6.23	7.49	14.42	14.45	16.50	5.74	5.96	6.05
S.5.Adenosyl. L.methionine.p. toluenesulfonate. salt	C00019	HMDB0001185	6.95	6.91	7.51	10.18	11.60	10.69	8.44	8.20	8.50	11.99	12.06	11.82
Serine	C00065	HMDB0000187	10.44	10.61	10.31	7.50	6.97	7.32	12.47	12.58	11.65	8.05	8.41	8.45
Stearoyl. L.carnitine	None	HMDB0000848	13.01	13.40	12.74	8.03	6.41	5.22	10.47	9.96	14.65	7.63	9.00	9.09
Succinic.acid	C00042	HMDB0000254	9.41	10.64	11.71	6.52	7.93	7.37	13.49	12.04	12.32	8.34	8.46	9.92
Taurine	C00245	HMDB0000251	8.75	8.77	8.25	9.14	8.94	8.85	9.97	9.93	10.58	10.37	10.54	10.28
Threonine	C00188	HMDB0000167	8.27	9.34	7.72	10.13	9.46	8.30	11.39	10.61	10.64	9.73	9.79	8.71
trans.4.Hydroxy. L.proline	C01157	HMDB0000725	8.15	8.45	8.21	9.27	8.93	8.40	11.10	9.70	11.16	9.93	10.47	9.78
UMP	C00105	HMDB0000288	9.24	8.89	8.92	9.25	8.92	7.84	10.60	10.96	9.47	12.29	11.04	10.56
Uracil	C00106	HMDB0000300	8.68	9.26	8.49	9.21	9.92	10.01	10.18	9.90	10.38	11.00	11.40	10.54
Urate	C00366	HMDB0000289	9.45	8.52	8.35	8.34	8.55	8.89	11.16	9.68	11.42	10.04	11.35	8.48
Uridine	C00299	HMDB0000296	10.63	11.25	10.13	8.90	9.37	7.95	13.55	13.05	12.65	8.60	8.17	8.19
Valeryl. L.carnitine	None	HMDB0013128	5.77	6.09	5.56	13.34	12.14	11.11	5.10	5.60	5.31	15.55	15.65	16.79
Xanthine	C00385	HMDB0000292	8.95	9.17	7.64	9.17	8.84	9.80	8.76	9.06	9.83	12.14	12.54	11.26

a; Compound entry in Kyoto Encyclopedia of Genes and Genomes
b; The Human Metabolome Database
nd; not done

Supplementary Table 2. Metabolites with significant differences

Name	Exp 1						Exp 2						Fold[a] change	P value[b]
	Scr			SiPLIN2			Scr			SiPLIN2				
	1	2	3	1	2	3	1	2	3	1	2	3		
S.5.Adenosyl.L. methionine.p.toluenesulfonate.salt	0.98	0.97	1.05	1.43	1.63	1.50	1.01	0.98	1.01	1.43	1.44	1.41	1.47	1.20E-07
N.Acetyl. L.aspartic.acid	0.97	1.11	0.92	1.30	1.36	1.37	1.00	1.05	0.95	1.71	1.52	1.58	1.47	4.93E-05
Methionine	1.06	1.01	0.93	1.27	1.34	1.46	1.06	0.91	1.04	1.14	1.35	1.32	1.31	1.07E-04
Citrulline	0.94	1.03	1.03	1.09	1.21	1.13	1.01	0.99	1.01	1.11	1.14	1.13	1.13	1.10E-04
Valeryl.L.carnitine	0.99	1.05	0.96	2.30	2.09	1.91	0.96	1.05	1.00	2.91	2.93	3.15	2.55	6.75E-04
Nicotinamide.hypoxanthine.dinucleotide.sodium.salt	0.93	0.92	1.15	1.27	1.60	1.34	1.16	1.08	0.77	1.27	1.61	1.40	1.42	8.52E-04
D.Tryptophan	1.03	1.01	0.96	1.37	1.27	1.32	1.01	0.99	1.00	1.20	1.19	1.09	1.24	1.54E-03
Uracil	0.99	1.05	0.96	1.04	1.13	1.14	1.00	0.97	1.02	1.08	1.12	1.04	1.09	1.93E-03
L.Carnosine	0.99	0.89	1.12	1.23	1.41	1.30	1.06	0.97	0.97	1.10	1.16	1.13	1.22	3.24E-03
Glutathione. oxidized.	0.98	1.04	0.98	1.14	1.18	1.14	1.04	0.97	0.99	1.42	1.27	1.45	1.27	4.49E-03
Hypoxanthine	1.08	1.01	0.91	1.41	1.58	1.59	0.97	0.99	1.03	2.58	2.88	2.58	2.10	8.42E-03
Xanthine	1.04	1.07	0.89	1.07	1.03	1.14	0.95	0.98	1.07	1.32	1.36	1.22	1.19	1.21E-02
L.Tyrosine	0.97	0.99	1.04	1.31	1.43	1.43	1.08	0.98	0.94	1.06	1.09	1.08	1.23	2.30E-02
IMP	0.90	1.07	1.03	1.06	1.26	1.20	1.00	1.05	0.95	2.01	1.92	1.78	1.54	2.31E-02
Glutamine	1.02	1.01	0.97	2.07	1.98	1.96	1.02	0.96	1.02	1.19	1.21	1.13	1.59	2.44E-02
DL.Leucine	0.76	1.07	1.16	1.35	1.53	1.57	1.05	0.94	1.01	1.10	1.11	1.00	1.28	3.52E-02
Glutathione. reduced.	1.08	1.00	0.92	1.10	0.96	1.02	0.96	0.99	1.05	1.22	1.27	1.21	1.13	4.23E-02
DL. lauroylcarnitine	1.08	1.08	0.84	0.96	0.99	1.33	0.90	1.01	1.09	1.20	1.36	1.33	1.19	4.59E-02
5-Deoxy.5. methylthio. adenosine	1.01	0.94	1.04	1.34	1.15	1.13	0.99	0.96	1.05	1.05	1.06	1.02	1.13	4.66E-02
N.acetyl.neuraminic.acid	1.06	0.98	0.96	0.55	0.59	0.60	1.01	1.02	0.97	0.54	0.52	0.55	0.56	6.73E-10
Serine	1.00	1.02	0.99	0.72	0.67	0.70	1.02	1.03	0.95	0.66	0.69	0.69	0.69	9.52E-10
L.Malic.acid	1.02	1.02	0.95	0.83	0.79	0.81	0.97	1.04	0.98	0.75	0.78	0.77	0.79	3.03E-07
D.Glucose.6.phosphate.potassium.salt	1.04	0.99	0.97	0.74	0.77	0.74	1.03	1.03	0.94	0.83	0.80	0.83	0.79	3.85E-06
L.Acetylcarnitine	0.99	1.09	0.92	0.75	0.68	0.73	0.92	1.06	1.02	0.61	0.60	0.59	0.66	9.54E-06
Succinic.acid	0.89	1.01	1.11	0.62	0.75	0.70	1.07	0.95	0.98	0.66	0.67	0.79	0.70	2.26E-05
Ribose.5. phosphate	1.12	0.99	0.89	0.71	0.57	0.69	0.95	0.96	1.09	0.38	0.39	0.40	0.52	5.40E-05
6-phosphogluconate	1.13	0.92	0.95	0.74	0.65	0.73	0.94	0.93	1.13	0.64	0.42	0.50	0.61	1.52E-04
Lactate	1.06	0.94	0.99	0.89	0.88	0.93	1.00	0.98	1.02	0.91	0.87	0.87	0.89	2.10E-04
CMP	0.95	0.84	1.21	0.61	0.77	0.63	1.00	1.02	0.98	0.81	0.75	0.66	0.71	6.15E-04
Uridine	1.00	1.05	0.95	0.83	0.88	0.75	1.04	1.00	0.97	0.66	0.62	0.63	0.73	1.04E-03
Stearoyl. L.carnitine	1.00	1.03	0.98	0.62	0.49	0.40	0.90	0.85	1.25	0.65	0.77	0.78	0.62	1.05E-03
Cytidine	1.00	1.01	0.99	0.80	0.87	0.79	0.99	0.95	1.06	0.61	0.58	0.60	0.71	1.93E-03

L.Carnitine	1.03	1.09	0.88	0.87	0.76	0.83	0.90	1.11	0.99	0.84	0.85	0.82	0.83	1.99E-03
Alanine	0.99	1.00	1.01	0.89	0.93	0.86	1.00	0.99	1.02	0.77	0.79	0.73	0.83	2.53E-03
NAD	0.99	1.03	0.98	0.95	0.98	0.90	1.05	1.01	0.94	0.91	0.86	0.84	0.91	5.35E-03
Butyryl. L.carnitine	0.98	1.05	0.97	0.98	0.90	0.97	1.02	1.02	0.96	0.92	0.88	0.87	0.92	6.06E-03
Adenosine	1.03	1.01	0.95	0.79	0.81	0.74	1.24	0.84	0.92	0.85	0.75	0.73	0.78	8.50E-03
Propionyl. L.carnitine	0.98	1.13	0.89	0.79	0.71	0.74	0.91	1.05	1.04	0.01	0.69	0.64	0.60	9.16E-03
Glycine	0.98	1.02	1.00	0.91	0.97	0.97	1.03	0.98	0.98	0.77	0.76	0.78	0.86	1.98E-02
Palmitoyl. L.carnitine	0.98	1.16	0.86	0.82	0.63	0.76	0.91	0.92	1.17	0.76	0.91	0.95	0.80	2.06E-02
Creatine	1.00	1.02	0.97	0.92	0.97	0.81	0.93	1.05	1.02	0.92	0.99	0.90	0.92	2.15E-02

Values are converted from supplementary table 1 taking the average of three scr as 1 for each experiment

a; Fold change= (Average of SiPLIN2 from Exp 1 and Exp 2)/ (Average of Scr from Exp 1 and Exp 2)

b; p value of student's t test comparing 6 sample of SiPLIN2 from Exp 1 and Exp 2 vs, 6 samples Scr from Exp 1 and Exp 2

Supplementary Table 3. Human primers used for Sybr green qPCR

	FORWARD	REVERSE
ACTB[ref9]	GAAGATCAAGATCATTGCTCCT	TACTCCTGCTTGCTGATCCA
HPRT1[ref9]	CCTGGCGTCGTGATTAGTGAT	AGACGTTCAGTCCTGTCCATAA
XBP1S [ref10]	TGCTGAGTCCGCAGCAGGTG	GCTGGCAGGCTCTGGGGAAG

ACTB; Actin, beta, HPRT1; Hypoxanthinephosphoribosyl transferase1, XBP1s; X-box binding protein, spliced form

Supplementary Table 4. Antibodies used for Western blots

guinea pig anti-ADFP	1:5000	ProSci[a]	Figure 1B
rabbit anti-GAPDH (14C10)	1:1000	2118, Cell Signaling Technology	Figure 1B, 9E, 9G
rodent anti-Total OXPHOS	1:1,000	ab110413, abcam	Figure 2H, 5H, 10H and supplementary figure 4E
Rabbit anti-ADFP	1:1,000	Ab52335, abcam	Figure 9E, 9G and supplementary 4B
Mouse anti-tubulin (TU-02)	1:3,000	sc-8035, Santa Cruz Biotechnology	Figure 2H, 10H
HRP-conjugated goat anti-guinea pig IgG	1:10,000	sc-2438, Santa Cruz Biotechnology	Figure 1B
HRP-conjugated mouse IgG kappa binding protein (m-IgGκ BP)-IgG	1:3,000	sc-516102, Santa Cruz Biotechnology	Figure 9E, 9G
HRP-conjugated mouse anti-rabbit IgG	1:10,000	sc-2357, Santa Cruz Biotechnology	Figure 1B, 2H, 5H, 9E, 9G, 10H and supplementary figure 4B, 4E
DyLight 800 goat anti-Mouse IgG (H+L)	1:10,000	SA5-35521, ThermoFisher	Figure 10H

a; Custom made by ProSci, Inc. using CDPQQSVVMRAVANLPLVSSTYDL, which is 7 to 28 amino acids of mouse Plin2 with addition of C at the beginning to increase the antigenicity.
HRP: horseradish peroxidase

Supplementary Table 5: Donor ID of human islets used

Data	Source		
	IIDP[a]	Alberta Institute Core[b]	Prodo[c]
Figure 9A-D			HP-17055<br>HP-17061<br>HP-17075
Figure 9E<br>Representative blot	SAMN08971735		
Figure 9E<br>Densitometry	SAMN08971735		HP-17040<br>HP-17055<br>HP-17075
Figure 9F, H	SAMN10737781<br>SAMN11476721<br>SAMN11483342<br>SAMN11864195		
Figure 9G<br>Representative blot	SAMN10977276		
Figure 9G<br>Densitometry	SAMN10977276<br>SAMN14120450<br>SAMN15400953<br>SAMN15579355	SAMN12044342<br>SAMN15239415	
Figure 9I, J	SAMN10737781<br>SAMN11476721<br>SAMN11483342<br>SAMN14132340<br>SAMN15400953<br>SAMN15579355	SAMN15239415	
Figure 10A	SAMN11476721		
Figure 10B-G	SAMN11476721<br>SAMN11483342<br>SAMN11864195<br>SAMN13938639<br>SAMN14120450<br>SAMN14132340<br>SAMN15400953<br>SAMN15579355		
Figure 10H<br>Representative blot	SAMN15400953		
Figure 10H<br>Densitometry	SAMN14120450<br>SAMN15400953<br>SAMN15579355	SAMN15239415	

a; Integrated Islet Distribution Program (https://iidp.coh.org)
b: Alberta Diabetes Institute Islet Core (https://www.epicore.ualberta.ca/isletcore/)
c; Prodo laboratories INC (https://prodolabs.com)

Supplementary Table 6: Donor and islet characteristics of human islets used

	Donor characteristics					Islet characteristics		
ID	Age (years)	Sex (M/F)	BMI (kg/m^2)	HbA1c	Cause of death	Islet isolation center	Purity (%)	Viability (%)
SAMN 08971735	50	M	32.3	5.4	Head trauma	The Scharp-Lacy Research Institute/IIDP	90	95
SAMN 10737781	66	M	27.2	4.7	Cerebrovasc ular/stroke	The Scharp-Lacy Research Institute/IIDP	95	95
SAMN 10977276	52	M	27.2	5.7	Cerebrovasc ular/stroke	Southern California Islet Cell Resource Center/IIDP	85	96
SAMN 11476721	50	M	32.8	6.0	Anoxia	The Scharp-Lacy Research Institute/IIDP	90	95
SAMN 11483342	52	F	39.8	6.4	Anoxia	University of Wisconsin/IIDP	92	98
SAMN 11864195	53	F	40.0	5.5	Cerebrovasc ular/stroke	University of Wisconsin/IIDP	92	98
SAMN 13938639	50	F	39.2	5.0	Cerebrovasc ular/stroke	Southern California Islet Cell Resource Center/IIDP	80	95
SAMN 14120450	37	M	31.9	5.6	Head trauma	University of Pennsylvania/IIDP	85	96
SAMN 14132340	31	M	27.0	5.2	Head trauma	Southern California Islet Cell Resource Center/IIDP	85	95
SAMN 15400953	54	F	24.5	5.7	Cerebrovasc ular/stroke	The Scharp-Lacy Research Institute/IIDP	90	95
SAMN 15579355	21	M	27.2	5.1	Head trauma	The Scharp-Lacy Research Institute/IIDP	85	95
SAMN 12044342	44	F	23.2	4.9		Alberta Institute Core		90
SAMN 15239415	31	F	20.3	4.8		Alberta Institute Core		95
HP-17040	46	M	27.6	5.4	Cerebrovasc ular/stroke	Prodo laboratories	90	95
HP-17055	41	M	28	5.3	Head trauma	Prodo laboratories	90	95
HP-17061	38	M	39	5.8	Anoxia	Prodo laboratories		
HP-17075	37	F	19.8	4.6	Anoxia	Prodo laboratories	95	95

References

1. McManaman JL, Bales ES, Orlicky DJ, Jackman M, MacLean PS, Cain S, et al. Perilipin-2-null mice are protected against diet-induced obesity, adipose inflammation, and fatty liver disease. *J Lipid Res.* 2013;54(5):1346-59.

2. Doliba NM, Liu Q, Li C, Chen J, Chen P, Liu C, et al. Accumulation of 3-hydroxytetradecenoic acid: Cause or corollary of glucolipotoxic impairment of pancreatic beta-cell bioenergetics? *Mol Metab.* 2015;4(12):926-39.

3. Roat R, Rao V, Doliba NM, Matschinsky FM, Tobias JW, Garcia E, et al. Alterations of pancreatic islet structure, metabolism and gene expression in diet-induced obese C57BL/6J mice. *PLoS One.* 2014;9(2):e86815.

4. Yamazaki M, Munetsuna E, Yamada H, Ando Y, Mizuno G, Murase Y, et al. Fructose consumption induces hypomethylation of hepatic mitochondrial DNA in rats. *Life Sci.* 2016;149:146-52.

5. Kang C, Xie L, Gunasekar SK, Mishra A, Zhang Y, Pai S, et al. SWELL1 is a glucose sensor regulating beta-cell excitability and systemic glycaemia. *Nat Commun.* 2018;9(1):367.

6. Cantor JR, Abu-Remaileh M, Kanarek N, Freinkman E, Gao X, Louissaint A, Jr., et al. Physiologic Medium Rewires Cellular Metabolism and Reveals Uric Acid as an Endogenous Inhibitor of UMP Synthase. *Cell.* 2017;169(2):258-72 e17.

7. Li B, Tang J, Yang Q, Li S, Cui X, Li Y, et al. NOREVA: normalization and evaluation of MS-based metabolomics data. *Nucleic Acids Res.* 2017;45(W1):W162-W70.

8. Chong J, Soufan O, Li C, Caraus I, Li S, Bourque G, et al. MetaboAnalyst 4.0: towards more transparent and integrative metabolomics analysis. *Nucleic Acids Res.* 2018;46(W1):W486-W94.

9. Li X, Yang Q, Bai J, Xuan Y, and Wang Y. Identification of appropriate reference genes for human mesenchymal stem cell analysis by quantitative real-time PCR. *Biotechnol Lett.* 2015;37(1):67-73.

10. van Schadewijk A, van't Wout EF, Stolk J, and Hiemstra PS. A quantitative method for detection of spliced X-box binding protein-1 (XBP1) mRNA as a measure of endoplasmic reticulum (ER) stress. *Cell Stress Chaperones.* 2012;17(2):275-9.
